# A close-up view on ITS2 evolution and speciation - a case study in the Ulvophyceae (Chlorophyta, Viridiplantae)

**DOI:** 10.1186/1471-2148-11-262

**Published:** 2011-09-20

**Authors:** Lenka Caisová, Birger Marin, Michael Melkonian

**Affiliations:** 1 Universität zu Köln, Biozentrum Köln, Botanisches Institut, Zülpicher Str. 47b, 50674 Köln, Germany; 2 Current Address: Institute of Botany v.v.i., Academy of Sciences of the Czech Republic, Dukelská 135, 379 82 Třeboň, Czech Republic, CZ; 3 Current Address: University of South Bohemia, Faculty of Science, Branišovská 31, 370 05 České Budějovice, Czech Republic, CZ

## Abstract

**Background:**

The second Internal Transcriber Spacer (ITS2) is a fast evolving part of the nuclear-encoded rRNA operon located between the 5.8S and 28S rRNA genes. Based on crossing experiments it has been proposed that even a single Compensatory Base Change (CBC) in helices 2 and 3 of the ITS2 indicates sexual incompatibility and thus separates biological species. Taxa without any CBC in these ITS2 regions were designated as a 'CBC clade'. However, in depth comparative analyses of ITS2 secondary structures, ITS2 phylogeny, the origin of CBCs, and their relationship to biological species have rarely been performed. To gain 'close-up' insights into ITS2 evolution, (1) 86 sequences of ITS2 including secondary structures have been investigated in the green algal order Ulvales (Chlorophyta, Viridiplantae), (2) after recording all existing substitutions, CBCs and hemi-CBCs (hCBCs) were mapped upon the ITS2 phylogeny, rather than merely comparing ITS2 characters among pairs of taxa, and (3) the relation between CBCs, hCBCs, CBC clades, and the taxonomic level of organisms was investigated in detail.

**Results:**

High sequence and length conservation allowed the generation of an ITS2 consensus secondary structure, and introduction of a novel numbering system of ITS2 nucleotides and base pairs. Alignments and analyses were based on this structural information, leading to the following results: (1) in the Ulvales, the presence of a CBC is not linked to any particular taxonomic level, (2) most CBC 'clades' sensu Coleman are paraphyletic, and should rather be termed CBC grades. (3) the phenetic approach of pairwise comparison of sequences can be misleading, and thus, CBCs/hCBCs must be investigated in their evolutionary context, including homoplasy events (4) CBCs and hCBCs in ITS2 helices evolved independently, and we found no evidence for a CBC that originated via a two-fold hCBC substitution.

**Conclusions:**

Our case study revealed several discrepancies between ITS2 evolution in the Ulvales and generally accepted assumptions underlying ITS2 evolution as e.g. the CBC clade concept. Therefore, we developed a suite of methods providing a critical 'close-up' view into ITS2 evolution by directly tracing the evolutionary history of individual positions, and we caution against a non-critical use of the ITS2 CBC clade concept for species delimitation.

## Background

The second Internal Transcriber Spacer (ITS2) is a fast evolving part of the nuclear-encoded rRNA operon, located between 5.8S and 28S rRNA genes. To obtain mature, functional rRNA molecules, the entire rRNA operon is transcribed as a single precursor rRNA, followed by complex excision processes of both ITS regions [1-3]. Similar to introns and non-transcribed spacer regions, the primary sequence of ITS2 appears highly variable, however, the excision process of the ITS2 RNA transcript (briefly termed'ITS2') requires certain secondary structure motifs, which seem to be conserved across most eukaryotes [4-6]. ITS2 usually folds into a clover leaf-like secondary structure with four helices, two of which show additional sequence/structure motifs that again appear to be essential for successful excision of ITS2 from the precursor rRNA molecule. In contrast to Helix1 and Helix 4, which are highly variable in sequence and length, Helix 2 is more conserved and almost always displays at least one pyrimidine-pyrimidine (UxU, UxC, CxC) mismatch [4,7]. Helix 3 is usually much longer than the other helices, and its apical region shows high sequence conservation, often including a four nucleotide motif (YGGY) [6,7]. This motif is close to the crucial cleavage site C2 where the degradation process of ITS2, i.e. the formation of the mature 5.8S and 28S rRNA, is initiated by a hitherto unidentified endonuclease [8-11]. Only in a few eukaryotes the ITS2 apparently deviates from these common features [6,12], or is absent altogether [13].

The presence of a stable and functionally important RNA secondary structure can be revealed by comparing homologous positions among different organisms, and searching for non-conserved, but co-evolving nucleotides, which maintain base pairing in the RNA transcript, thus indicating the presence of intra-molecular RNA helices [4,14,15]. Generally, RNA helices can retain base pairing by two evolutionary processes, double-sided changes (i.e. co-evolution), and single-sided changes. In the former, a substitution on one side of the helix (e.g. G → C), which would disrupt base pairing, can be compensated by changing the nucleotide at the opposite side (i.e. C → G). The whole double-sided change (G-C → C-G) is called Compensatory Base Change (CBC; [4,14]). The existence of the non-canonical 'wobble' base pair (G-U), which is thermodynamically stable in RNA molecules, allows even single-sided changes that perfectly retain base pairing, and are accordingly named hemi-Compensatory Base Change (hCBC; e.g. G-U → G-C; [15,16]).

For two reasons ITS2 is thought to be an excellent marker for molecular phylogenetic studies, especially at lower taxonomic levels. Obviously, the highly divergent and fast-evolving ITS2 can discriminate among closely related organisms, which otherwise display almost identical sequences, e.g. in the conserved rRNA genes. This explains the frequent use of ITS2 for calculation of lower-level phylogenetic trees in many eukaryotic lineages [e.g. [17-23]]. In addition, ITS2 data have been used to predict the ability to interbreed successfully, thereby determining the limits between 'biological' species and populations [20,24,25]. The latter approach, introduced by Coleman and coworkers, consists basically of a pairwise comparison of ITS2 secondary structures from closely related organisms, considering only compensatory changes within ITS2 helices. Computing presence/absence of even a single Compensatory Base Change (CBC) in the conserved regions of helices 2 and 3 of ITS2 revealed a correlation with incompatibility/ability to sexually cross [25,26]. In contrast, changes in the less conserved regions (e.g. in helices 1 and 4) as well as hCBCs in the conserved parts did not correlate with interbreeding ability. Thus, Coleman [25] defined a group of organisms without any CBC in conserved ITS2 regions (i.e. in helices 2 and 3) as a CBC clade, which is distinguished from other CBC clades by at least one CBC in these regions. In addition, a group of organisms producing compatible gametes that can form zygotes was named Z clade [25]. Although members of different CBC clades apparently always fall into different Z clades, which are isolated by reproduction barriers such as inability of gamete fusion or other pre-zygotic isolation mechanisms, it is still possible that the members of the same CBC clade are unable to mate, and thus fall into two or more Z clades [15,27]. Moreover, a single CBC clade/Z clade is not necessarily equivalent to one 'biological species', defined by its fertile offspring, because a zygote may be unable to develop further due to post-zygotic barriers, e.g. failure to perform meiosis. In summary, a CBC clade corresponds to one or more Z clades, which itself may contain one or more 'biological' species.

Most described species have been defined solely on the basis of structural characters, and may be labeled 'morphospecies'. What is the relation of CBC clades, Z clades, and 'biological' species to previously described morphospecies? Unfortunately, no general rule can be applied here, as e.g. previously recognized by Coleman [26]. As one extreme case, morphologically identical organisms, classified as a single taxonomic species, represent one CBC clade containing multiple Z clades (e.g. *Chlamydomonas allensworthii *[28] or are a composite of several CBC clades and even more Z clades (e.g. *Pandorina morum *[29]. We may designate such cases cryptic species complexes (= type C in [26]). At the other extreme, morphologically diverse organisms, classified as different species or even genera, can successfully interbreed, and then belong to the same Z clade as well as CBC clade (e.g. Hawaiian silverswords - *Argyroxipium*, *Dubautia*, *Wilkesia *[30]; and genera of the Altingiaceae - *Liquidambar*, *Altingia *[31]), and may be regarded as hybridization events (= type A in [26]).

It has nevertheless been concluded that among potential mates, increasing ITS2 divergence is correlated with decreasing potential for mating and zygote formation [26]. Since there is no obvious functional link between ITS2 sequence and the process of gamete fusion, the observed correlation between CBCs and inability to cross has been explained by either similar or faster evolutionary rates of genes that control gamete interactions, compared to the rate of CBC-type changes in conserved ITS2 regions [25,26].

Therefore, it appears necessary to study the evolution of CBCs in paired ITS2 regions during recent and ancient diversification processes, and to estimate the frequency of these events in relation to mating barriers and the origin of new species. Regarding the first aspect, it is currently unclear whether CBCs usually evolve via two simultaneous changes on both sides of a helix, or instead represent the sum of two changes that occurred at different times, either as a series of two consecutive hCBC-type substitutions, or involving a non-paired intermediate state. It is further unknown whether CBC/hCBC rates and frequencies are similar throughout ITS2 helices, or whether these parameters are unequally distributed among ITS2 base pairs due to CBC/hCBC hotspots or CBC/hCBC silencing. Finally, regarding the importance especially of ITS2 CBCs for molecular taxonomic concepts, it appears surprising that the phylogeny of CBC-type changes usually plays no role in such analyses, whereas in other phylogenetic and taxonomic investigations, application of cladistic principles, i.e. strict distinction between plesiomorphic and apomorphic character states, is a commonality. In fact, CBCs are mostly visualized phenetically, i.e. as a pair-wise comparison between sister species [e.g. [20,21,32-35]]. Similarly, the homoplasy background of CBC-type substitutions in ITS2, i.e. presence of reversals, parallelisms, and convergences, has not been analyzed so far.

In the present contribution, we investigated these questions in detail, selecting the green algal order Ulvales (Ulvophyceae, Chlorophyta) as a case study. The Ulvales provide (1) many available ITS2 and 18S rDNA sequences, (2) data from crossing experiments, (3) morphological and taxonomic diversity, and (4) distribution over freshwater, brackish, and marine habitats. We reconstructed a consensus ITS2 secondary structure for the Ulvales, and introduced a new numbering system based on positional homology. By mapping all evolutionary changes that occurred in ITS2 helices across the investigated Ulvales, we found that CBC clades mostly do not correlate with the level of 'biological' species, and are often paraphyletic assemblies (here named CBC grades) rather than genuine monophyletic (holophyletic) clades. Furthermore, our analyses revealed CBCs and hCBCs as clearly independent evolutionary processes, which only rarely occurred in the same ITS2 base pairs, largely characterized different branches in the phylogenetic tree, and displayed different homoplasy background levels. In particular, we found no evidence that would support the hypothesis that CBCs evolved through two consecutive hCBCs.

## Results

### Folding methods for ITS2

**Figure 1:**
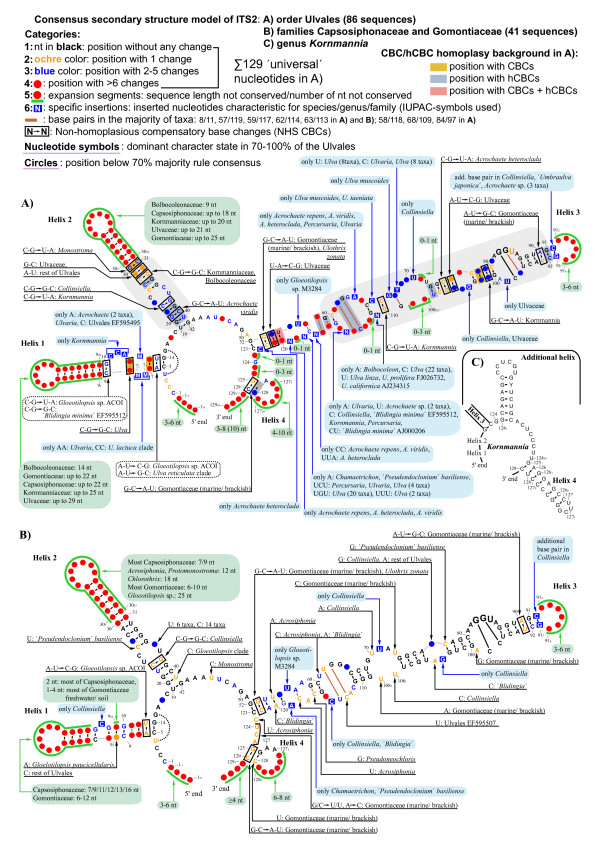
**Consensus secondary structure models of ITS2 in the Ulvales**. **A) **Consensus ITS2 diagram based on 86 sequences covering five families (Kornmanniaceae, Bolbocoleonaceae, Ulvaceae, Capsosiphonaceae and Gomontiaceae). **B) **ITS2 consensus of the Capsosiphonaceae and Gomontiaceae (41 sequences analyzed), showing extremely high conservation. Nucleotide letters shown in both ITS2 diagrams (**A, B**) refer to the most frequently occurring character states among the analyzed taxa, obtained via 70% majority rule consensus sequences. Positions without 'dominant' character state among the investigated Ulvales were integrated as circles, or flagged as expansion segments. Invariable positions were drawn in black, whereas for variable positions, the conservation/variability level was quantified by the number of evolutionary changes during the diversification of the Ulvales, and indicated by various colors (nucleotides and/or circles in ochre, blue, or red). 129 positions were present in all studied Ulvales, and were used for a 'universal' numbering system of ITS2 positions. The 'non-universal' positions were labeled with subscript numbers, combined with the previous 'universal' position number. Gray shades indicate the conserved parts of helices 2 and 3 [6]. Several comments in the Figure refer to non-homoplasious CBCs (black frames), inserted positions characteristic for selected taxa, and the length variation of expansion segments. The CBC/hCBC homoplasy background in the ITS2 diagram (**A**) is indicated by ochre/blue/pale pink shades with the restriction to positions with CBCs/hCBCs/CBCs+hCBCs respectively. **C) **Simplified ITS2 diagram of *Kornmannia *displaying a unique, additional helix between helices 3 and 4.

Using the programs MFold [36] and RNAstructure [37,38], homologous regions of the ITS2 sequence were generally folded as comparable secondary structural motifs, and revealed four universal helices present in all 86 Ulvales analyzed here (Helix 1 to 4 in Figure 1). Comparison of these universal helices across taxa identified several base-paired positions that retained pairing by covariation (compensatory base changes, CBCs; e.g. C-G ⇒ A-U), or by a change in only one position (hemi-compensatory base changes, hCBCs; e.g. C-G ⇒ U-G). Numerous CBCs and hCBCs confirmed the 'genuine' structure of ITS2, and rejected artificial folding patterns.

Using another tool for ITS2 secondary structure generation, i.e. 4SALE [39,40] combined with the ITS2 Database III [41], resulted in conflicting folding patterns for different taxa, and the only common feature among these folds was the presence of four helices (Additional file 1). However, these helices were often generated from non-homologous sequence regions, and thus could not be compared across taxa. A check of 'template models' from the ITS2 Database III revealed only a few ulvophyte ITS2 folds that, except for some discrepancies in Helix 3, corresponded to our consensus secondary structure model (e.g. ITS2 of *Ulva fasciata*; Additional file 1). Although most other 'template models' of ulvophytes showed a correctly folded Helix 2, the remaining helices contained several folding errors, as is obvious from clearly homologous sequence motifs in non-comparable secondary structural placements (see Additional file 1).

### Consensus secondary structure model of ITS2

The ITS2 showed only moderate length variation across the Ulvales, ranging from 171 (uncultured *Urospora *AJ626846) up to 205 (*Acrochaete *sp. EF595429) or 235 nucleotides (*Kornmannia*; see below). The high degree of secondary structure conservation allowed the unambiguous alignment of most ITS2 positions, and generation of a consensus secondary structure model of the ITS2 in the Ulvales (Figure 1). This model included a variability map, i.e. all positions were classified into different categories: (1) 100% conserved nucleotides, (2) highly conserved positions with only one unique change within the Ulvales, (3) moderately conserved positions with 2-5 changes, (4) variable positions with > 6 changes, (5) expansion segments (regions without length conservation, e.g. terminal loops of helices), and (6) specific insertions, i.e. positions that were present in only some taxa. In addition, comments in Figure 1 provide an overview about taxonomic entities with unique evolutionary changes (categories 2, 3), and with ITS2 length variations (categories 5, 6).

Within the Ulvales, five ITS2 regions were well conserved in primary sequence and secondary structure: (1) the first 2-3 base pairs of Helix 1, (2) the spacer between Helix 1 and Helix 2, (3) the basal part of Helix 2, containing 10 base pairs, (4) the spacer between Helix 2 and Helix 3, and (5) the apical part of Helix 3 (excluding the terminal loop) covering ca. 18-23 base pairs (Figure 1A). The remaining ITS2 motifs, including Helix 4 and the apical part of Helix 1, were much less conserved.

One major subclade of the Ulvales, encompassing the families Capsosiphonaceae and Gomontiaceae (often referred to as Acrosiphonaceae and Ulothrichaceae, respectively) was characterized by an even higher conservation of ITS2 positions, and therefore, a separate consensus secondary structure model was designed for these two families (Figure 1B). Among these families, the consensus model revealed high conservation for several ITS2 regions, which were rather variable among other Ulvales, e.g. the complete Helix 3 (compare Figure 1A and 1B).

One genus, *Kornmannia*, was exceptional due to the presence of an additional helix, located between Helix 3 and Helix 4, and an unusually long Helix 4 (Figure 1C).

### Introduction of a numbering system for ITS2 positions

The ITS2 consensus structure diagram (Figure 1A) provided the opportunity to introduce a novel numbering system of ITS2 nucleotides for unambiguous positional descriptions of base pairs, CBCs, hCBCs, and indels. Figure 1A revealed 129 homologous characters that were present in all Ulvales investigated here. These 129 'universal' characters served as the backbone of the new numbering system. In contrast, non-universal positions (variability categories 5 and 6 in Figure 1A) were labeled with subscript numbers (1, 2, 3...) combined with the 5' -preceding 'universal' nucleotide number (see Figure 1A). For example, 'universal' nt 7 at the 5´end of Helix 1 is followed by two non-universal nucleotides that were present only in *Ulvaria *and the *U. lactuca *clade, and these positions were named 7_1 _and 7_2 _(Figure 1A). The additional helix unique for *Kornmannia *was labeled in the same way (Figure 1C). As universal position number 'one', we arbitrarily designated the first moderately conserved (i.e. category 3) nucleotide of ITS2, since the 5'-end region was non-conserved in sequence and length (labeled 1_-1_, 1_-2 _... 1_-6 _in Figure 1A).

### ITS2 and 18S rDNA phylogeny of the Ulvales

**Figure 2:**
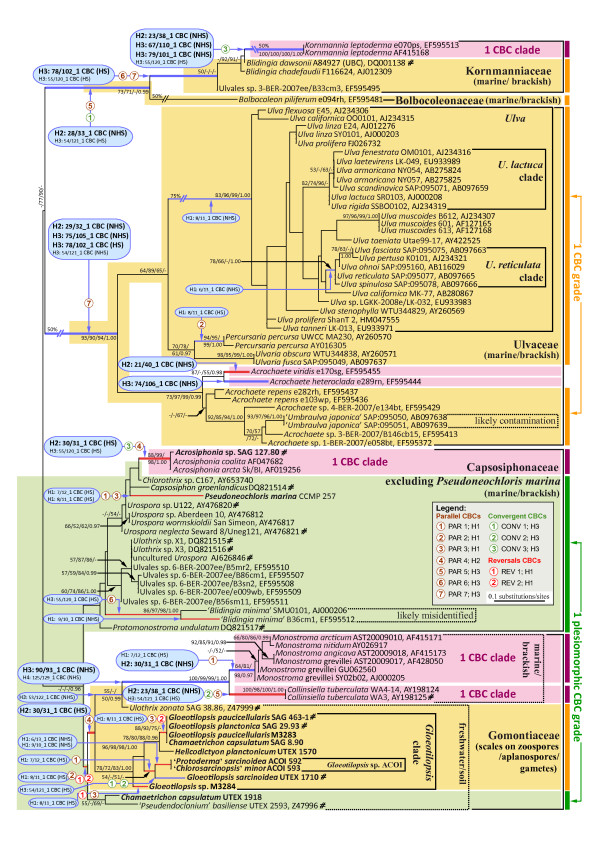
**Evolution of CBCs in paired ITS2 nucleotides mapped upon the ITS2 phylogeny of the Ulvales**. All compensatory base changes (CBCs) accompanied by appropriate Helix (H) and numbers of positions (by specific nucleotide numbers) were linked to the nodes/branches where they evolved. CBCs that occurred in the conserved regions of helices 2 and 3 (H2+3_CBCs) were shown in bold and in larger font size and their corresponding branches were depicted in bold as well. Branches in blue are characterized by CBCs and hCBCs, whereas branches in red color have CBC support exclusively. Only those taxa, which formed a terminal, monophyletic clade and were not differentiated by any CBC in the conserved parts of ITS2 helices (H2+3_CBCs), were here designated as a CBC clade, and were highlighted in pink background color. In contrast, taxa lacking distinguishing H2+3_CBCs, which formed non-monophyletic assemblies in the phylogenetic tree, were designated as 'CBC grades', and shaded in orange color. Note that all CBC grades contained nested CBC clades. Typically, a CBC clade/grade can be traced back to a common ancestor (basal branch) characterized by synapomorphic H2+3_CBCs, except for one 'plesiomorphic CBC grade' (green color) characterized merely by shared plesiomorphies in helices 2 and 3 of ITS2. CBCs either evolved as unique (non-homoplasious; NHS) or homoplasious synapomorphies (HS). CBCs were homoplasious due to parallelisms (PAR 1-7), convergences (CONV 1-3) and/or reversals (REV 1-2), and all these changes were mapped upon the tree (encircled numbers). The tree topology was based on 152 aligned ITS2 characters from 86 taxa analyzed by maximum likelihood (ML). The branch separating the Capsosiphonaceae/Gomontiaceae from the remaining Ulvales was used for rooting the tree. Four interrupted branches have been graphically reduced to 50% or 75% of the original length. Significances at branches from left to right are bootstrap percentages (ML, NJ, and MP) and Bayesian posterior probabilities. Newly determined sequences (12) are in bold (for accession numbers see Additional file 7). Taxa/strains with hash mark (#) were also analyzed in the 18S rRNA phylogeny (Additional file 2).

ITS2 provided 152 alignable characters for phylogenetic analyses of 86 taxa in the Ulvales (Figure 2). As an additional control, we performed phylogenetic analyses of an 18S rDNA data set of 74 Ulvales using 1702 characters (Additional file 2). The taxon sampling in both data sets was largely non-congruent since 18S rDNA + ITS2 data were available for only 15 strains (taxa marked with hash (#) in Additional file 2 and Figure 2). Five families of the order Ulvales and *Pseudoneochloris marina *(a non-resolved single branch) were well represented in both alignments, whereas the families Chlorocystidaceae and Phaeophilaceae (Additional file 2) were missing in the ITS2 data set.

Although both phylogenies cannot be directly compared, the absence of conflicting branching patterns suggested that the phylogenetic signal in ITS2 was sufficient to resolve most relationships among the Ulvales correctly. Among basal branches (family and genus levels) we observed almost no conflict case (exception: *Pseudoneochloris*). However, overall support values differed considerably between 18S rDNA and ITS2 phylogenies owing to the lower number of aligned ITS2 characters - all basal branches of families gained high support by 18S rDNA data, whereas the corresponding branches in the ITS2 phylogeny were usually non-supported (Additional file 2, Figure 2). The only exception was the family Ulvaceae that gained high support by ITS2 also. At the genus and species level, several possible cases of conflict between 18S rDNA and ITS2 analyses were observed, e.g. relationships among the genera *Acrochaete*, *Umbraulva*, *Ulvaria *and *Percursaria*. However, a reliable comparison between these phylogenies was not possible due to the non-congruent taxon sampling, and some likely misidentified taxa or presence of contaminations (e.g. '*Blidingia minima*' as a member of the family Capsosiphonaceae or *Acrochaete *spp. growing on '*Umbraulva japonica*' as an epiphyte in Figure 2).

### Compensatory Base Changes (CBCs) and hemi-Compensatory Base Changes (hCBCs)

**Figure 3:**
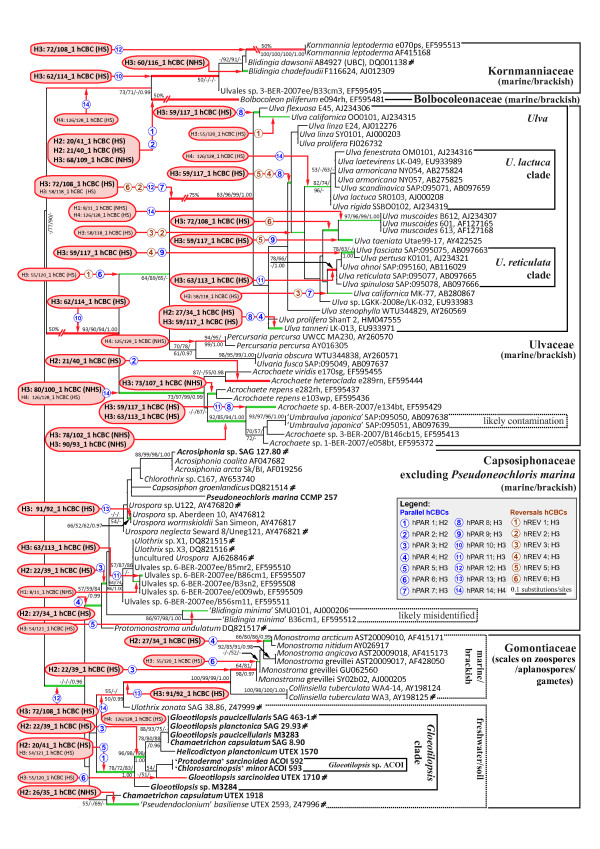
**Evolution of hCBCs in ITS2 base pairs in the Ulvales**. Hemi-compensatory base changes (hCBCs) referring to conserved parts of helices 2 and 3 were shown in bold and in larger font size and their corresponding branches were illustrated in bold. Branches in red are characterized by hCBCs and CBCs, whereas branches in green color have hCBC support exclusively. Encircled numbers were used to indicate all hCBC-type parallelisms (hPAR 1-14) and reversals (hREV 1-6); hCBC-type convergences were not found in the ITS2 of the Ulvales.

To identify all positions that co-evolved as double/single-sided changes in an ITS2 helix with conservation of base pairing (CBCs/hCBCs) within the Ulvales, an exhaustive apomorphy search was performed among paired ITS2 characters (Additional file 3, 4). In total, 38 CBCs were revealed over all helices and only 15 of these were discovered in the relatively conserved regions of helices 2 and 3 (gray shades in Figure 1A) and were collectively termed 'H2+3_CBCs' (bold and larger font size in Figure 2 and Additional file 5). In the same way, all 51 hCBCs have been depicted in Figure 3 (hCBCs in bold and large font size). From the 15 H2+3_CBCs only one (Helix 3: 75/105 in Ulvaceae) was adjacent to a bulge and this is the only example in which a pairing might have moved over one nucleotide on one strand (slippage). Regarding hCBCs, 12 hCBCs from 34 H2+3_hCBCs were located next to a bulge. Furthermore, two different categories of CBCs/hCBCs could be distinguished: CBCs/hCBCs that uniquely characterized a single branch/clade within the Ulvales (Non-Homoplasious Synapomorphies - NHSs; NHS CBCs are illustrated in black frames in Figure 1), and CBCs/hCBCs that evolved in a homoplasious manner (HS; see below).

All 38 CBCs and 51 hCBCs, including the homoplasious changes, were mapped upon the phylogenetic tree inferred from the ITS2 sequences comparisons (Figures 2, 3), and were assigned to 24 and 41 clades/branches, respectively (colored in Figures 2, 3) where they evolved (the total number of tree branches is: 105 [Figures 2, 3]). Interestingly, CBCs and hCBCs were distributed over both terminal and internal branches on the tree (Figures 2, 3).

### CBC clades and CBC grades

For CBC clade-based concepts of species delimitation, either Helix 3 alone (the relatively conserved 30 base pair region in proximity to the GGU motif; [26]) or the relatively conserved regions of helices 2 and 3 [e.g. [6]] have been considered as essential. A group of organisms characterized by the absence of any CBCs in these conserved pairing regions of ITS2 has been defined as a CBC clade sensu Coleman [[25], page 6]. In total 15 H2+3_CBCs were found in the Ulvales (comprising 50 currently accepted species [42]) and were assigned to 11 branches/clades flagged by blue/red colors in bold in Figure 2. All 15 H2+3_CBCs and their appropriate branches were analyzed for matching the CBC clade definition sensu Coleman [25]. In summary, only two of the 15 H2+3_CBCs were mapped on species-branches within species-rich genera (*Acrochaete heteroclada*, *A. viridis*; Figure 2).

Furthermore, it has been revealed that four of 11 branches defined monophyletic CBC clades that differed from all 'outgroup' taxa by the presence of at least one H2+3_CBC (clades shaded in pink in Figure 2; e.g. *Monostroma*, *Acrosiphonia*). Other major clades were also characterized by H2+3_CBCs, but contained nested subclades that again gained novel synapomorphic H2+3_CBCs. In these cases, the nested (monophyletic) subclades formed genuine CBC clades, whereas the remaining taxa (major clade minus nested CBC clades) formed non-monophyletic assemblies of organisms, which were not distinguished by any CBC-type difference in helices 2 and 3 (shaded in orange or green colors in Figure 2). In other words, we found the majority of the Ulvales within non-monophyletic groups that clearly failed to meet the classical definition of CBC clades (see above). Because the term CBC clade is restricted to ITS2 clades (i.e. monophyletic lineages) lacking of any H2+3_CBCs among its members [25], we herein introduce the term 'CBC grade' (orange color in Figure 2), defining a non-monophyletic assemblage of organisms without any H2+3_CBC among its members. Four of five CBC grades were differentiated from all non-members by at least one H2+3_CBC, i.e. delineated from derived taxa (= nested CBC clades) as well as 'outgroup' taxa. As an example, all Ulvaceae to the exclusion of the derived members *Acrochaete heteroclada *and *A. viridis *(37 taxa in Figure 2) represented a single paraphyletic CBC grade, well differentiated from other Ulvales by three H2+3_CBCs, and from *A*. *heteroclada *and *A. viridis *by one H2+3_CBC, respectively. Similarly, the Kornmanniaceae formed a CBC grade to the exclusion of *Kornmannia*, which itself formed a terminal CBC clade.

As an exception, one of the CBC grades [Capsosiphonaceae + Gomontiaceae excluding three nested CBC clades (*Acrosiphonia, Monostroma, Collinsiella *) and one nested CBC grade (*Gloeotilopsis *clade + *Ulothrix zonata*; Figure 2), 20 taxa marked in green background in Figure 2] was devoid of any synapomorphic CBC in the ITS2 helices. These 20 taxa shared plesiomorphic character states for all ITS2 base pairs in the conserved regions of helices 2 and 3, and represented a 'plesiomorphic CBC grade', merely united by absence of any synapomorphy of the H2+3_CBC type.

**Figure 4:**
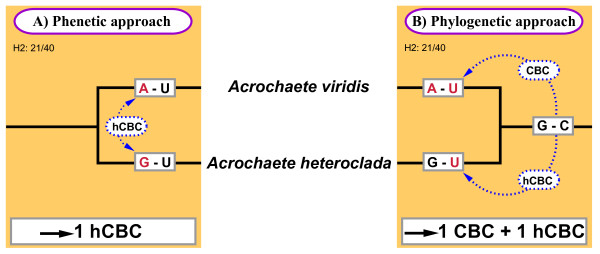
**Phenetic versus phylogenetic approach of species delimitation of two taxa of *Acrochaete***. **A) **Phenetic approach, i.e. pair wise comparison without consideration of the plesiomorphic status of the base pair 21/40 in the conserved region of Helix2 (H2) revealed that *A. viridis *and *A. heteroclada *differ by only one hCBC (A-U vs. G-U, respectively). In contrast, **B) **a phylogenetic approach taking the ancestral status (G-C) of the respective base pair into consideration resulted in the difference of one CBC (G-C → A-U) + one hCBC (G-C → G-U) between these two taxa. Whereas the phenetic approach would,- according to the CBC clade concept, regard *A. viridis *and *A. heteroclada *to belong to a single CBC clade (and potentially the same species), the phylogenetic approach showed *A. viridis *and *A. heteroclada *as two separate species. Base pair 21/40 as well as its plesiomorphic status (both in gray boxes) are mapped on the branches; and the evolving CBC/hCBC are indicated by blue dashed arrows.

Usually, CBC substitutions between sister taxa are identified and quantified by pairwise comparison of their ITS2 secondary structures, i.e. by a phenetic rather than a phylogenetic approach. In one case (base pair 21/40 in Helix 2 of *Acrochaete heteroclada *and *A. viridis*) it became obvious that such a phenetic comparison can be misleading when the third relevant 'taxon', i.e. the common ancestor of *A*. *heteroclada *and *A. viridis*, is not taken into consideration (for details, see Figure 4). Whereas the phenetic method would suggest that both taxa differ merely by a single hCBC, the synapomorphy search revealed presence of a CBC plus one hCBC, and thus identified *A. viridis *and *A*. *heteroclada *as two different species.

### CBCs, hCBCs, branch lengths and evolutionary rates

To correlate the frequency of CBCs and hCBCs in ITS2 helices with the evolutionary rates of the branches where they occurred [measured by branch lengths (evolutionary steps), considering base-paired positions exclusively], these parameters were recorded for all 105 branches in the ITS2 phylogeny (Figure 2) and plotted as diagrams (Additional file 6). The majority (79% for CBCs, 58% for hCBCs) of shorter branches (lengths of up to nine evolutionary steps) lacked any CBC and/or hCBC, and thus showed non-compensatory changes exclusively (base pair ⇔ non-pair). Thus, branch lengths appeared neither strictly correlated with the number of CBCs, nor hCBCs. However, when only those branches with one and two CBCs were considered, the number of CBCs seemed weakly correlated with branch lengths up to about 13 evolutionary steps (Additional file 6A). Among the long branches (lengths > 13), the relation to CBCs was unclear due to the low sampling (only three branches), and the 'exceptional' long branch of *Bolbocoleon *without any CBC. Only the remaining two long branches (Ulvaceae and *Kornmannia*) showed the highest observed numbers of CBCs (four, respectively), indicating some correlation with branch lengths. This correlation, however, appeared non-linear but instead resembled a hyperbolic saturation curve. To analyze saturation, we calculated the CBC vs. branch length ratio (CBC_R, considering only branches with > 0 CBCs), and clearly found negative correlation between CBC_R (blue squares in Additional file 6A) and branch lengths. As an example, all four evolutionary steps that constituted the short branch of *Gloeotilopsis *sp. ACOI co-evolved as two CBCs (CBC_R 100%), whereas in *Kornmannia*, only eight out of 21 (CBC_R 38%) evolutionary steps made up four CBCs.

Regarding hCBCs, the relation to branch lengths was unclear due to the generally low number of hCBCs per branch, i.e. mostly one, rarely two (only seven branches), or three (only *Bolbocoleon*, Additional file 6B). Among clades with > 0 hCBCs, the hCBC vs. branch length ratio (hCBC_R) was similarly decreasing between the short branches (hCBC_R 33-100%, for branch lengths up to three) and the longer branches where hCBC_R approached 4.8% for *Kornmannia *(one hCBCs vs. 21 evolutionary steps; blue squares in Additional file 6B), again indicating saturation.

### Evolutionary relationship between CBCs and hCBCs, and their parallelisms, convergences, and reversals

When CBCs and hCBCs were mapped upon clades/branches of phylogenetic trees using an exhaustive synapomorphy search, their occurrence was clearly non-correlated with each other (compare Figures 2 and 3). Only 11 branches shared CBCs + hCBCs (branches in blue + red, respectively), whereas 12/29 branches displayed CBCs/hCBCs exclusively (branches in red/green in Figures 2, 3 respectively). Branches with exclusive CBC support (red branches in Figure 2) represented eight terminal branches as well as four internal divergences. Similarly, their hCBC counterparts (green branches in Figure 3) were distributed over 11 terminal and 18 internal branches.

**Figure 5:**
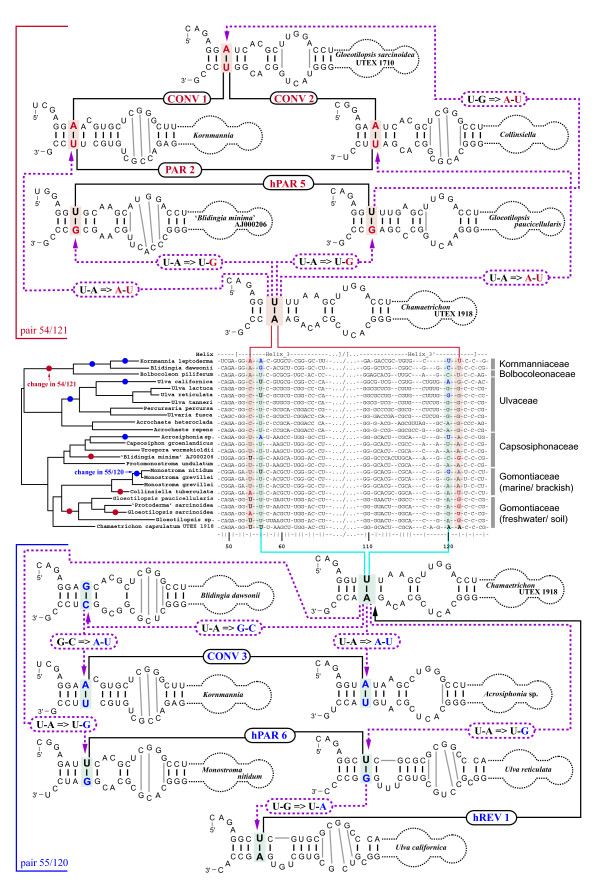
**Parallelisms, convergences, and reversals in ITS2 base pairs, as shown by selected examples**. All three cases of homoplasious changes occurred in ITS2 pairs 54/121 and 55/120 in the basal part of Helix 3 in the Ulvales. For all taxa in the simplified phylogram (derived from Figure 2), sequences of the basal part of Helix 3 are presented as an alignment, and linked to secondary structure diagrams highlighting the homoplasious character evolution of base pair 54/121 (in red color, above alignment) and pair 55/120 (in blue color, below alignment). Dashed arrows in violet were used to indicate the evolutionary direction of homoplasious changes. In the phylogram, branches/clades in which homoplasious substitutions occurred were marked by red/blue circles. Homoplasious changes were abbreviated as before (Figures 2, 3).

The synapomorphy search strategy further revealed all existing homoplasious changes of ITS2 base pairs, i.e., all parallelisms, convergences, and reversals of CBCs and hCBCs in the Ulvales (Additional files 3, 4). These homoplasies were also mapped on the tree topologies, associated with branches (Figures 2, 3). As a parallelism, we regarded identical evolutionary changes in unrelated lineages, starting from the same plesiomorphic character state, and applied a simple numbering system, i.e. PAR1, PAR 2 etc. for parallel CBCs, and hPAR1, hPAR2 etc. for hCBCs. A given parallelism can refer to up to five unrelated lineages (e.g. hPAR 14; Figure 3, Additional file 4). Convergences differed from parallelisms by starting from different ancestral character states, e.g. G-C ⇒ A-U and U-A ⇒ A-U (labeled CONV in Figures and Additional files). A change back to a plesiomorphic character state, i.e. a reversal, was labeled REV for CBCs, and hREV for hCBCs. Figure 5 provides selected examples for these homoplasious changes that occurred in pairs 54/121 and 55/120 in the basal part of Helix 3, by showing alignments, folding diagrams, and evolutionary changes.

As a result, the homoplasy background underlying CBC-type changes differed profoundly from homoplasy frequences found for hCBCs in the Ulvales. Regarding parallelisms, 16 of 38 total CBCs (42%) evolved as parallelisms, occurring in seven ITS2 base pairs (PAR 1-7), whereas among all 51 hCBC, 38 (75%) represented parallelisms in 14 ITS2 pairs (hPAR 1-14; Additional file 4). The much higher homoplasy level of hCBCs was also mirrored by the remaining homoplasy types. Among the reversals, only two cases of the CBC-type were found, which both occurred in the same highly variable base pair in Helix 1 (8/11; REV 1-2, Additional file 4). In contrast, we found six hCBCs that represented reversals towards the ancestral character state (hREV 1-6; Additional file 4). We even found a twofold switch between ancestral and derived character states via hCBC-type reversals. As a synapomorphy in base pair 58/118, C-G changed to U-G in the genus *Ulva*, followed by a reversal in one major *Ulva *subclade (U-G ⇒ C-G = hREV 2) and a more recent second reversal in *U. californica *AB280867 (C-G ⇒ U-G = hREV 3; Figure 3, Additional file 4). Notably, convergences were confined to the CBC category exclusively, and occurred three times in two ITS2 base pairs (CONV 1-3; Figure 5 and Additional file 4).

**Figure 6:**
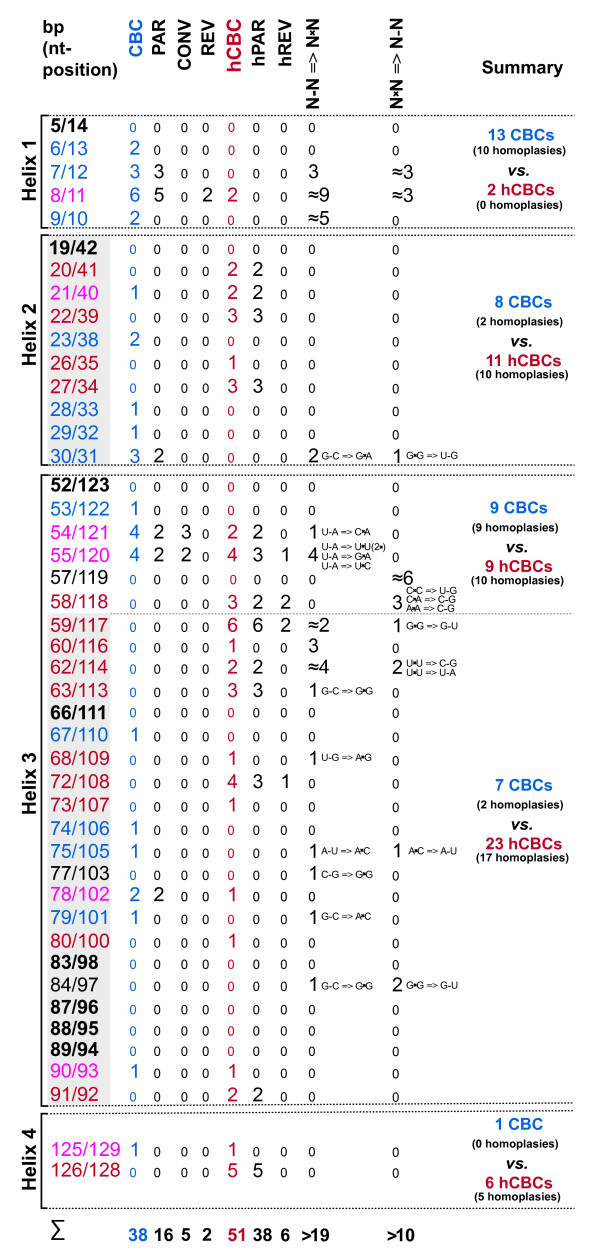
**Occurrence/frequency of substitutions of ITS2 base pairs, and homoplasious changes, mapped upon ITS2 helices**. Occurrence and frequency of all compensatory (i.e. CBCs, hCBCs) and non-compensatory substitutions of ITS2 base pairs, and homoplasious changes (parallelisms, convergences, reversals), mapped upon ITS2 helices. Eight invariant base pairs are in bold; the conserved parts of helices 2 and 3 are in gray. Base pairs displaying CBCs + hCBCs are indicated in pink, pairs evolved exclusively via CBCs are in blue, and pairs developed solely via hCBCs are drawn in red. Non-compensating substitutions (N-N ⇔ N×N) were especially frequent in homoplasious positions of Helix 1 and in two pairs of Helix 3 (55/120, 62/114). For Helix 1 and 3 base pairs, the total number of non-compensatory changes cannot be estimated precisely due to their high substitution frequency.

To further investigate the relation between CBCs and hCBCs, their frequencies of occurrence and frequencies of homoplasies were mapped upon all universal base pairs of ITS2 helices (Figure 6). Again, CBCs as well as hCBCs were unequally distributed, i.e., non-correlated. CBCs occurred frequently in Helix 1 and the basal part of Helix 3. In these 'variable' regions, CBCs evolved with a high homoplasy background including all recovered REV and CONV-type homoplasies, whereas in the conserved parts of helices 2 and 3 very few homoplasious CBC-type changes occurred (only PAR; Figure 6). Hemi-CBCs showed the opposite tendency - low frequency in Helix 1, but much higher frequencies of occurrence in the remaining regions of ITS2 helices, including the conserved regions (for details, see Figure 6). Except for Helix 1, the homoplasy background underlying hCBCs was equally high throughout ITS2 base pairs (Figure 6).

Addressing individual base pairs in Figure 6 revealed even more than a non-correlation between CBCs and hCBCs - actually, co-occurrence of CBCs and hCBCs in the same base pair was exceptional. In the Ulvales, only seven base pairs displayed CBCs + hCBCs (pink), whereas 27 pairs either evolved exclusively via CBCs (12, blue) or exclusively via hCBCs (15, red in Figure 6).

**Figure 7:**
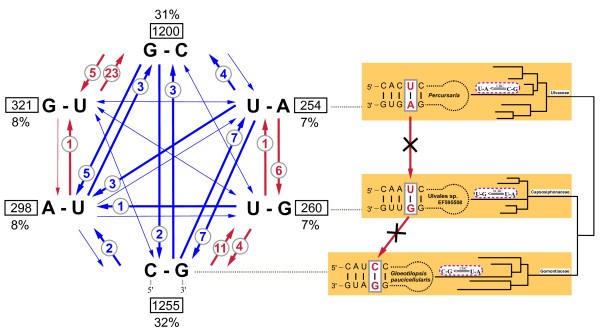
**Diagram showing evolutionary changes of base pairs, and their frequency/occurrence in ITS2 of the Ulvales**. All possible evolutionary changes between canonical (C-G, A-U) and 'wobble' (G-U) base pairs in RNA molecules, and their frequency of occurrence in the entire ITS2 are illustrated. Base pairs are given in 5'-3' orientation, referring to their placement in a helix. Arrows indicate the evolutionary direction of substitutions. ITS2 changes, which were found in the Ulvales are indicated by bold arrows, accompanied by frequencies (encircled numbers), whereas changes that were not existent in the analyzed taxa (frequency 0) are shown as thin arrows. CBCs are shown in blue, hCBCs are in red. Obviously, hCBCs (especially the G-C ⇔ G-U type) occurred more frequently than CBCs. From the frequency of base pairs (number in black boxes) and its percentage (number below black boxes) it is evident that there was a strong selection towards the GC/CG category. Note that the given frequencies of base pairs are confined to extant taxa. The illustration of Helix 1 with pair 8/11 highlighted refers to the hypothesis that a CBC of the U-A ⇔ C-G type may have evolved via two consecutive hCBC steps (see Results). Note, however, that the taxa shown are unrelated, indicated by the simplified trees, which provide no support for this hypothesis.

It may be assumed that CBCs of the C-G ⇔ U-A category may often have originated by two consecutive hCBC-type substitutions, i.e. following the pathway C-G ⇔U-G ⇔ U-A. Therefore, we investigated the contribution of hCBCs to the observed CBCs in ITS2 helices of the Ulvales, and surprisingly found no single case supporting the above-mentioned theoretical pathway. In our case study, this result can neither be explained by low frequency of the C-G ⇔ U-A category, nor of the respective single hCBCs (C-G ⇔U-G, and U-G ⇔ U-A). In Figure 7, we listed canonical (C-G, A-U) and 'wobble' (G-U) RNA base pairs, taking into account their orientation in the helix, and the frequency of all possible single (hCBC) and double (CBC) substitutions that retain base pairing in the Ulvales. Obviously, almost all above-mentioned changes occurred during ITS2 evolution of the Ulvales, mostly with high overall frequencies, except for 5'-U-G-3' ⇒ U-A (only one case), 5'-A-U-3' ⇒ G-U (only one case) and the reverse change (5'-G-U-3' ⇒ A-U, no case; Figure 7). Especially, the theoretical pathway 5'-U-A-3' ⇒ U-G ⇒ C-G appeared well supported by high frequencies of the individual hCBC categories, as well as the frequently found direct CBC change (U-A ⇒ C-G). However, most of the individual hCBCs referred to different ITS2 base pairs, and thus cannot be regarded as intermediates in the evolution of a CBC. Only one base pair in Helix 1 (position 8/11) showed all character states required for the hypothetical hCBC pathway (see above) in different taxa (U-A in *Percursaria*, U-G in e.g. Ulvales sp. EF595508, and C-G in e.g. *Gloeotilopsis paucicellularis*; Figure 7). Since in all phylogenetic analyses these taxa formed unrelated terminal branches within three families, rather than forming a single phyletic series (Figure 2), they gained their character states in position 8/11 independently (via CBCs in two cases), and therefore cannot be considered as an example of a CBC that was gained by two consecutive hCBCs (see Figure 7). In summary, hCBCs evolved with a different homoplasy background, changed on different branches in the phylogeny of the Ulvales, largely preferred different ITS2 base pairs than those yielding CBCs, and did not contribute to CBCs observed in ITS2 helices of the Ulvales.

## Discussion

In the present contribution we developed a suite of methods to gain 'close-up' insights into ITS2 evolution that may guide future studies of ITS diversification in general. Therefore, we propose a general strategy for studies of ITS evolution and phylogeny, starting with the minimal requirements of the data set. ITS sequences differ from most other molecular markers by their low primary sequence and length conservation, and only the common intra-molecular folding pattern of their RNA transcripts, i.e. their secondary structure, allows comparative investigations. The correctly folded secondary structure is fundamental not only for improving the alignment [43-48], but also for building the alignment itself (especially in case of variable markers such as ITS2) as well as for identifying and detecting synapomorphies. In fact, the secondary structure is a prerequisite for all conclusions derived from the phylogenetic analyses. Even with many available sequences, deciphering the 'genuine' secondary structure is a demanding procedure, since the initial secondary structure folding process of a single ITS2 sequence (e.g. via MFold) often yields several alternative folds, and must be performed with ITS2 sequences from as many closely/distantly related taxa as is possible, to select the common folding pattern, substantiated by occurrence of CBCs and hCBCs [4,49]. To simplify this analysis, an alternative, standardized procedure has been developed in which a novel ITS2 sequence is automatically compared to > 110.000 sequences in the ITS2 Database III with known secondary structures as a reference [46,50]. However, for selected ITS2 sequences of the Ulvales, we obtained clearly false folding patterns using the ITS2 Database III. This is especially surprising since the authors described their criteria for how to evaluate the quality of secondary structure models, e.g. presence of four helices with conserved helix length distribution, and a UGGU motif near the 5' site apex of Helix III [51]. However, some of the artificial 'reference' ITS2 structures of the Ulvales were in conflict with these criteria. Moreover, even structures that comply with the standards may often represent artifacts, as shown here for the Ulvales. As a conclusion, the time-consuming manual approach to identify the common ITS2 secondary structure for a selected group of organisms as done here cannot be abbreviated by a semi-automated procedure without significant loss of accuracy.

Fortunately, the ITS2 sequences of the order Ulvales proved to be an almost ideal model for comparative structural and phylogenetic studies. These sequences were unusually well conserved in length, and contained many, almost invariable sequence motifs, which allowed high-quality alignments. Sequence conservation allowed integration of more than 80 ITS2 sequences of the Ulvales, which together represented five families, within a single alignment - so far a unique case in the algae where an ITS2 data set is usually confined to a single family or genus. Furthermore, most ITS2 folds (using MFold or RNAstructure) spontaneously favored the same overall secondary structure, which corresponded well with already known ITS2 features in other green algae [4,6]. Hallmarks of this common secondary structure, as e.g. the start/end of the four helices, and the spacers between helices, could easily be related to highly conserved sequence motifs in the ITS2 alignment. Even the most highly divergent ITS2 regions that were not alignable by manual sequence comparison showed excellent secondary structure conservation that allowed an unambiguous alignment across all Ulvales, except for the apical parts of the four helices. In consequence, each column in the alignable ITS2 regions represents a single homologous character, which applies not only for the paired positions but also for single-stranded spacer and internal loop regions.

To achieve an Ulvales-wide system to identify and number ITS2-nucleotides as a statement of positional homology, all unambiguously aligned positions were either classified as 'universal', i.e., present across all Ulvales, or 'non-universal', i.e. existing in only some Ulvales and thus being subject to insertion/deletion events. Only the first group of nucleotides were given 'universal' position numbers (1-129), allowing a clear nomenclature of e.g. ITS2 base pairs. These universal positions covered the whole range between invariable, moderately variable, and highly variable characters. To specify the conservation status of individual positions, usually a majority rule consensus is generated across the taxa investigated, e.g. a character that is G in 80 out of 100 taxa is termed '80% conserved' [4,16,52]. Here, we instead used the absolute number of changes in the evolution of a given character as a more appropriate measure of its degree of conservation. As an example, both positions of base pair 29/32 changed only once in the evolution of the Ulvales in the common ancestor of a taxon-rich family, the Ulvaceae. Thus, by simple majority rule consensus these characters would be regarded as 'less than 55% conserved', whereas our evolutionary measure (one change) clearly reveals their high conservation.

Following clarification of homology, universality, nomenclature, and the degree of variation of ITS2 characters, summarized in consensus secondary structure diagrams, all character state changes (substitutions) of each position could be investigated in detail to deduce the rules under which ITS2 evolved towards its current diversity. As a method, the previously developed synapomorphy search procedure [52] automatically generated a complete inventory of all substitutions of ITS2 positions within the Ulvales, and in addition, precisely identified the branches in the phylogenetic tree where these substitutions occurred. Since the most interesting questions regarding ITS2 evolution are related to the paired positions in the double-stranded helices, the resulting list of single-character evolutionary changes was analyzed manually to trace the evolution of all known base pairs for (1) co-evolution by maintaining base pairing via CBC, and (2) single-sided changes retaining pairing via hCBC. The result of this screen is an overview of all recent CBC- or hCBC-type changes underlying terminal branches, as well as changes that characterize basal divergences in the phylogeny of the Ulvales. Especially the latter point marks a difference to other studies where ITS sequences of extant taxa are compared without consideration of evolutionary changes that led to these sequences [53-55].

Are CBC frequencies proportional to the overall sequence divergence? To analyze this question, previous investigators [56,57] plotted the ITS-distances between pairs of extant taxa against the number of CBCs, and found similar relations: CBC-frequencies (maximally 8-9 CBCs) are increasing from low to medium distance values, while for highly diverging pairs of sequences the number of CBCs is relatively small, indicating saturation. Surprisingly, this distribution was analyzed by linear regression methods and then characterized as 'linear proportional relation' [56]. In the present study, synapomorphy searches revealed all CBCs, and precisely identified the branches on which they occurred. These data allowed a phylogenetic rather than a statistical approach, i.e. by plotting CBC frequencies versus the length (determined for paired sites only) of the respective internal or terminal branch. For the Ulvales, we also found a saturation-type relation between CBC frequencies and branch lengths, with the CBC vs. branch length ratio (CBC_R) being negatively correlated with branch lengths. In their study on Myrtaceae [57], the authors assumed 'unobserved' substitutions for the distant sequence comparisons, i.e. reversals, as one reason for the low number of observed CBCs, and also noticed that CBCs actually occur at relatively few sites in ITS molecules. We fully confirmed the latter phenomenon - out of 45 'universal' base pairs in ITS2, only 19 pairs underwent CBC-type changes throughout the entire order Ulvales. In other words, the limited number of sites that can per se evolve via CBCs may be the major reason for the unexpectedly low number of CBCs in divergent branches or taxa. As an example, the long branch of *Kornmannia *(21 substitutions), which could theoretically involve up to 10 CBCs, actually shows CBCs at only four sites. As an alternative explanation for the observed saturation in divergent branches or taxa, a high rate of 'unobserved' CBCs may be assumed, i.e. CBCs, which were immediately reverted towards the ancestral state. However, the synapomorphy analysis/mapping approach performed here allowed precise quantification of CBC-type reversals throughout the Ulvales: among 38 CBCs, we found only two reversals. Therefore, it appears very unlikely that high rates of 'unobserved' CBCs contributed to CBC saturation in the Ulvales. All these data suggest that CBCs represent a complex evolutionary process, which at higher divergence levels is constrained by available sites in ITS2 rather than depending simply on overall sequence divergence.

It is usually assumed that a CBC cannot evolve by two simultaneous substitutions, given the low evolutionary rates of most paired positions in ITS2 [57,58]. Instead, a CBC may have evolved by two single-sided changes within a short time, and usually, the 'wobble' pair (G-U) is assumed as intermediate, suggesting the series A-U ⇔ G-U ⇔ G-C that represents two consecutive hCBCs [58-64]. As an alternative scenario, the intermediate stage may comprise mismatching nucleotides (e.g. A-U ⇔ AxC ⇔ G-C). Although the '2x hCBC → CBC' scenario seems attractive, it only applies for one case of CBC (A-U ⇔ G-C), and not to any of the remaining observed CBC categories (e.g. A-U ⇔ U-A/U-G/C-G). A popular approach to address this question is to determine frequencies of the respective changes. In the Ulvales, hCBCs of the A-U ⇔ G-U type as well as the G-U ⇔ G-C type were observed at high numbers, suggesting that in fact CBCs may have evolved via two subsequent hCBC-steps. However, such a summarizing view of overall substitution rates, which is often applied as the only source of evidence [e.g. [57]], can be misleading for two reasons. First, these hCBCs may have occurred at different positions (see below), and second, even if these hCBCs referred to the same ITS base pair, they may have evolved independently in organisms that do not form a phyletic series. In fact, our synapomorphy analysis readily revealed that almost all pairs of hCBCs, which could theoretically form a 2-step CBC, occurred in different ITS2 positions, and already this spatial separation within the ITS2 molecule makes any causal relation between CBCs and hCBCs highly unlikely. Only in a single case, both hCBCs required for a full 2-step CBC mapped upon the same ITS2 position in Helix 1 (Figure 7). However, the respective taxa were unrelated to each other, highlighting that both hCBCs emerged as independent evolutionary events that did not converge towards a CBC. The simple formula 2x hCBC → CBC can at best be regarded as an exceptional scenario, which, however, could not be demonstrated in the Ulvales. In contrast to the misleading conclusions derived from statistical methods, the specific reconstruction of the phylogenetic history of ITS2 base pairs via synapomorphy analysis resolved this question.

Are CBCs and hCBCs equally distributed over ITS2 positions, or can one recognize distinct positional preferences? In fact, only seven pairs in the entire ITS2 molecule displayed both CBCs and hCBCs, whereas all remaining pairs appeared 'specialized' to either category of change. Already this simple observation is difficult to reconcile with the notion that the majority of CBCs followed a '2x hCBC → CBC' pathway.

Taken together, a hCBC appears to be a stable substitution, suggesting that the 'wobble' pair (G-U) is not at a disadvantage compared with 'canonical' base pairs [63,65,66]. In other words, when a canonical pair underwent a hCBC that lead to G-U, there was no selection pressure in favor of an immediate second hCBC restoring a canonical pair. In the Ulvales, we found similar preferences for both directions of hCBCs: 23 hCBCs of the canonical → 'wobble' pair type, and a comparable number (28) of the 'wobble' → canonical pair type. Comparisons of models of RNA sequence evolution, using ITS data from angiosperms, also suggested absence of strong selection against non-canonical base pairs [57,64]. Interestingly, the evolutionary behavior of the 'wobble' pair is strongly biased in the Ulvales: we observed only a single hCBC of the G-U/U-G → A-U/U-A type, versus 27 hCBC in the G-U/U-G → G-C/C-G categories. A similar bias has been reported for some angiosperm families [57,64]. It seems attractive to explain such a bias in substitution rates by unequal frequencies of G-C/C-G (31/32%) and A-U/U-A pairs (8/7% in the Ulvales), as e.g. done by [57]. However, this conclusion is illegitimate (see below), and we favor another explanation, regarding functional constraints underlying a 'wobble' pair (for specific features of G-U, see [e.g. [66-69]]. The thermodynamic stability of A-U/U-A is more or less comparable to G-U/U-G, whereas the G-C/C-G pairs contribute much more to the stability of a helix [58,66,70,71]. Thus, G-U/U-G → A-U/U-A changes may be comparatively neutral compared to G-U/U-G → G-C/C-G changes, which may be under positive selection in the Ulvales. As a suggestion, exchanges towards G-C/C-G pairs could improve ITS2 folding stability [72] when an organism is undergoing specialization to habitats with higher temperatures, and perhaps, the fast-evolving hCBC pathways (G-U/U-G → G-C/C-G) allow rapid ecological adaptation processes, in contrast to two-step CBC-type changes.

How did double-sided CBCs in ITS2 actually evolve? We favor a 2-step scenario that involves a non-pair as a short-living intermediate, i.e. N-N → N×N → N-N. In contrast to the '2x hCBC → CBC' scenario, this pathway holds for all CBC categories (22; blue arrows in Figure 7). At least for base pairs under functional constraints, it should be assumed that any spontaneous single-sided substitution leading to a non-pair is disadvantageous, with impaired ITS2 folding and excision characteristics [73]. This event will usually lead to strongly reduced fitness or even extinction of the mutant genotype [65,72]. Alternatively, mutants may escape extinction by intragenomic rRNA homogenization, which reverts the mutation and thus restores ITS2 functions and fitness [74]. With respect to extant organisms, extinction of mutants as well as rRNA homogenization processes cannot be readily investigated. However, we may be able to recognize selection against non-pairs in the double-stranded backbone of ITS2 helices, by comparison of non-compensating changes (N-N ⇔ N×N) versus overall frequencies of CBCs and hCBCs [75]. In fact, disruption of pairs (N-N → N×N) and restoration of pairing (N×N → N-N) both occurred at much lower frequencies (ca. 19 and 10 cases, respectively, within the Ulvales; uncertain cases in highly variable pairs were ignored) than CBCs and hCBCs (38 and 51 cases, respectively). Several of the conserved pairs even evolved exclusively by compensating changes, without any non-pairs. In the apical part of Helix 3, however, we found a few 'exceptional' positions that were almost universally paired, but evolved towards non-pairs within suprageneric clades (e.g. pair 79/101) or even whole families (pairs 68/109 - Ulvaceae, 75/105 - Kornmanniaceae and Bolbocoleonaceae, 84/97- Ulvaceae). How is it possible that the mismatch status remained stable over long periods of time? All these 'exceptional' non-pairs are surrounded by several conserved pairs, which, we suspect, in combination lead to strong thermodynamic stability of this helix [72]. Therefore, a few isolated non-pairs in Helix 3 do apparently not reduce fitness and viability of the respective organisms, since e.g. the three families listed above belong to the ecologically most successful green algae in marine and coastal environments [42,76].

Our data regarding Helix 2 provide the strongest evidence of selection against mismatch pairs - among 10 universal base pairs, nine were invariably double-stranded in all Ulvales and evolved exclusively by CBCs and hCBCs. Only the most variable pair 30/31 located just before the expansion region showed a few cases of mismatch. It should be noted that the two- dimensional shape of Helix 2 is regarded as a highly conserved 'hallmark' of the ITS2 core structure, i.e. a basal stem comprising about five base pairs, followed by a short internal loop (bulge) consisting of 1-2 pyrimidine-pyrimidine mismatches, and an apical stem+loop region [4,43]. Experimental changes of this secondary structure by mutagenesis leads to failure in ITS2 excision at the transcript level, and especially, introduction of even one additional non-pair in the stem region is sufficient to prevent efficient pre-RNA processing [9]. This corresponds well with our investigations in the Ulvales - such a change is perhaps not viable. However, only the basal pair of Helix 2 is invariant in the order, whereas all remaining pairs evolved at moderate rates, and - except pair 30/31 - lacked changes that interrupt base pairing. Although it might initially seem paradoxical, we assume that especially in these cases CBCs may have originated via non-paired intermediate steps, which in most cases were rapidly eliminated by natural selection (extinction). As a rare event, a lethal mismatch pair regained the essential base pairing by a second substitution, which must have occurred within a short time frame. As an example, the C-G → G-C CBC in pair 23/38 in Helix 2 may have evolved via short-living CxC or GxG mismatch state.

To substantiate our hypothesis that in ITS2 CBCs and hCBCs follow different evolutionary rules, we further investigated their homoplasious changes, i.e. parallelisms, convergences, and reversals. Fortunately, the problem to distinguish these three types of homoplasy was readily achieved by our approach of direct mapping of all substitutions in ITS2 base pairs, in contrast to indirect statistical methods, e.g. calculating a homoplasy index [15,18]. As a first insight, parallelisms seem to be the most frequent case of homoplasy in ITS2, followed by reversals and convergences. Interestingly, parallelisms and especially reversals occurred much more frequently in the hCBC category. Considering the only slightly higher number of hCBCs (51) versus CBCs (38), we observed twice the number of parallelisms (38 versus 16), and even a threefold increase of hCBC-type reversals (6 versus 2; Figure 6). The remaining homoplasy category, i.e. convergence, shows the opposite tendency: we found five cases of CBC-type convergences, but no such event among hCBCs (Figure 6). This appears surprising, since there are only two possible pathways for hCBC-type convergences (A-U → G-U ← G-C, and U-A → U-G ← C-G), and most of these individual substitutions happened rather frequently (Figure 7). However, all these individual substitutions referred to different base pairs in ITS2, and therefore did not contribute to any hCBC-type convergence. What is the reason for the higher rate of CBC-type convergences? The explanation may be the higher number of possible pathways, since every base pair can directly originate via CBCs from four other pairs (Figure 7). As an example, A-U can theoretically evolve from G-C, U-A, U-G, or C-G. Notably, all these changes were found in the Ulvales (Figure 7) and in some cases referred to the same ITS2 position, thus leading to the observed CBC-type convergences (Additional file 4).

Since CBCs and hCBCs showed clear positional preferences (see above), it is not surprising that their homoplasies are also spatially separated in the ITS2 molecule. Among 17 homoplasious positions, only two showed CBC- as well as hCBC homoplasies (Figure 6). Interestingly, the most conservative regions of the ITS2, i.e. the conserved parts of Helix 2 and 3, were both characterized by very low frequencies of CBC-type homoplasies accompanied by unusually high rates of hCBC homoplasies (Figure 6). This phenomenon might explain why several authors have restricted their conclusions to (1) these conserved parts of ITS2, and (2) to CBCs. Obviously, most CBCs in the conserved regions are non-homoplasious changes, and thus offer informative molecular signatures, which unambiguously characterize taxa and clades (including CBC clades). In contrast, hCBC are usually considered as taxonomically meaningless (genotypes differing by one hCBC may even be able to mate), and this is mirrored by e.g. elevated homoplasy levels even in the conserved regions, and very high substitution rates.

Can we explain the observed substitution rates of CBCs and hCBCs in the ITS2 with empirical frequencies of the respective base pairs? It might appear logical to assume that a high frequency of a given base pair should correlate with a high rate of substitutions leading to that base pair. Within the Ulvales, G-C and C-G are the most frequently occurring base pairs in ITS2 (31 and 32%, respectively), whereas the four remaining pairs were comparatively rare, each counting for only 7-8% (Figure 7). Assuming a frequency-substitution rate correlation, we should observe the highest substitution rates for 'frequent ⇔ frequent' CBCs (G-C ⇔ C-G), lower rates for 'frequent ⇔ rare' interchanges (e.g. C-G ⇔ U-A), and the lowest substitution rates for the category 'rare ⇔ rare' (e.g. U-A ⇔ A-U). Our data clearly reject such a correlation, and rather show almost complete independence between frequency and substitution rates. For example, a direct 'rare → rare' CBC (U-A → A-U) shows the same rate as C-G → G-C from the 'frequent → frequent' category. Clearly, the highest observed substitution rates were found among the 'frequent ⇔ rare' interchanges, and this holds for the highest CBC-rates (C-G ⇔ U-A) as well as the highest hCBC rates (C-G → U-G, G-U → G-C).

How can we explain that substitution rates are obviously independent of frequencies? First, several base pairs in ITS2 are essential for proper secondary structure folding, and thus are under strict functional constraints. Not surprisingly, several strong G-C and C-G pairs contribute to ITS2 stability, and thus are conserved or even invariant, as shown in the ITS2 secondary structure diagram (Figure 1), explaining the unexpectedly low number of observed changes. However, there is also a general reason why frequencies cannot be correlated with substitution rates - observed frequencies apply to sequences of extant taxa only, whereas substitution rates refer to ancient as well as recent evolutionary changes. This means, that a single early occurring change, mapped upon a deep branch in the phylogenetic tree, will affect several descendent taxa and will thus considerably influence the base pair frequency distribution among recent taxa. In contrast, a recent substitution, mapped upon shallow or terminal branches, changes the base pair frequency of only few or even single taxa, with almost no effect on the observed overall frequencies.

As an example, in the Ulvales and also in angiosperms [57], the 'wobble' pairs G-U/U-G display much higher substitution rates with G-C/C-G than with A-U/U-A (see above). [57] argued that this bias in substitution rates is simply the result of the several fold higher frequencies of G-C/C-G versus A-U/U-A. For the above-mentioned reasons, this argument is inconclusive, and we instead propose functional constraints under adaptive processes as a possible explanation for the observed bias (see above).

What is the significance of ITS2 for taxonomy and species definition in the Ulvales? So far, the ITS2 molecule has only rarely been used as marker for phylogenetic analyses in the Ulvales, except in studies of single genera (*Acrochaete *- [77]; *Acrosiphonia *- [78]; *Blidingia *- [e.g. [79,80]]; *Collinsiella*/*Monostroma *- [81]; *Gloeotilopsis *- [82]; *Ulva *- [e.g. [23,83-88]]; *Ulvaria *- [89]; *Urospora *- [90]. As a first surprise, ITS2 proved to be well alignable across the entire order due to its high structural conservation and low sequence length divergence, and thus allowed reconstructions of the phylogenetic branching pattern even above the level of the sampled families. To test whether the ITS2 tree is accurate, it was compared with a phylogeny derived from 18S rDNA data that covered a similar, albeit not identical, set of taxa, and this comparison revealed only a few conflicting branching patterns (see Results). Thus, ITS2 is an exceptionally informative phylogenetic marker in the Ulvales (see also [91]), especially with respect to the relatively low number of alignable positions, and in future should be analyzed in combination with congruent data sets of other genes.

However, the most spectacular evolutionary aspect regarding ITS2 concerns its potential to predict sexual compatibility (intercrossing) among closely related organisms, thereby defining the level of 'biological' species. One of the most recent proposals is that any CBC in the ITS2 is informative, and when two ITS2 sequences differ by at least one CBC, they likely represent two species [56]. Although the predicted ITS2 secondary structure in the Ulvales shows a high degree of conservation, we found it very difficult, sometimes impossible or at least subjective to align the highly variable regions (red circles surrounded by green line in Figure 1). Applying the proposal by Müller et al. [56], variations in ITS2 lengths (as is observed in many taxa) would automatically result in the recognition of more species, an untenable situation. We therefore favour the more conservative proposal by Coleman [25,26] which refers to the presence of at least one CBC between two organisms in the conserved regions of ITS2 predicting a failure to sexually cross, i.e. these organisms represent two different species. Ideally, CBCs should have evolved at (1) approximately the same rate in sister lineages, and (2) at approximately the same or slightly slower rates than genes that control gamete compatibility. As a consequence, the 'first' CBCs should appear at about the same time, associated with shallow divergences in the phylogenetic tree, and should define several parallel clades (CBC clades sensu Coleman) that might correspond to 'biological' species. In this scenario, those branches where 'first' CBCs occurred could be connected by a single vertical line as e.g. shown in a cartoon phylogenetic tree [26]. In the Ulvales, we found that none of these 'ideal' assumptions is fulfilled.

Clearly, many 'first' CBCs in the Ulvales are not associated with shallow branches at the level of 'biological' species, but instead mapped upon deep divergences representing the levels of genera, families, or even higher taxonomic levels. Only a few taxonomic species were equivalent to single CBC clades, e.g. *Collinsiella tuberculata*. Most CBC clades (sensu Coleman) within the Ulvales are therefore based on deep-branching CBCs, and each of them contains up to about 30 taxonomic species in several genera. Analysis concentrating on the ITS2 region of the Volvocaeae revealed a remarkable correspondence between CBC clade, Z clade and species (e.g. *Gonium pectorale*), [25]. Is it, therefore, possible that each of these comprehensive CBC clades in fact represents only a single species, containing a diverging population of several morphotypes that are still able to cross? Unfortunately, the crossing capability of most species of the Ulvales analyzed here has not been investigated, but the limited evidence available may already address this question. Species of *Ulva *are well separated from each other by gametic mating barriers, as e.g. studied in detail for the same strains of *U. ohnoi*, *U. reticulata *and *U. fasciata *that were investigated here [92]. These three species form one of many subclades within the large CBC clade sensu Coleman that includes the entire genus *Ulva *as well as most other members of the family Ulvaceae. Further observations regarding morphological organization [e.g. [76,93-100]], ultrastructural characterization - e.g. presence/absence of scales on zoospores/aplanospores/gametes [82,101-113] and type of habitat e.g. [42,76] in other Ulvales lead to the same conclusion. For example, the macroalgae *Protomonostroma *(foliose, marine) and *Capsosiphon *(tubular thallus, marine), as well as the branched filamentous *Chamaetrichon *(square-shaped scales on zoospores, freshwater) and several unbranched filamentous microalgae (e.g. *Urospora*, no scales, marine) are not differentiated by a CBC in the highly conserved regions of helices 2 and 3.

In summary, genes controlling gamete compatibility as well as genes involved in structural differentiation apparently evolved much faster than most CBCs in the ITS2 of the Ulvales.

The scattered, non-synchronous distribution of CBCs has another, unexpected consequence. Several major CBC clades, which are based on ancient CBC events, contain nested CBC clades that originated by more recent CBCs. Thus, only the latter category is monophyletic, whereas the major CBC clades, deeply rooted in the phylogenetic tree, usually form paraphyletic groupings, here termed CBC grades. In the Ulvales, only a few taxa fall into one of the four 'genuine' CBC clades, whereas most taxa are distributed among five comprehensive CBC grades. In other words, the absence of a CBC in the highly conserved regions of helices 2 and 3 does not imply the presence of a monophyletic group nor is indicative of a close relationship (i.e. at the species level) among the taxa that share this trait. It remains to be determined whether non-synchronization of 'first' CBCs and thus predominance of CBC grades is a special feature of the Ulvales, or is widely distributed among eukaryotes.

Mapping all CBCs on the phylogenetic tree is the only method to distinguish between 'genuine' CBC clades and CBC grades. Coleman [29] already mapped CBCs in helices 2 and 3 of ITS2 upon the phylogeny of *Pandorina *isolates, similar to our approach, and to our knowledge this is still the only published reference. Although most members of *Pandorina *analyzed formed CBC (monophyletic) clades, the tree revealed the presence of CBC grades that contained isolates which are less closely related to each other than isolates that are excluded from the grade - because of the presence of a specific CBC (e.g. PmU879 + PmNoz3923/PmKiev). Unfortunately, ITS2 comparisons including CBC-concepts are commonly performed in a more simple way, i.e. by pairwise comparison between two taxa [e.g. [22,34,53,54,114-118]]. This 'phenetic' approach usually does not consider the phylogenetic history of CBC-type substitutions (plesiomorphic vs. apomorphic), and for different reasons it can lead to wrong conclusions (see Results). In the case of distantly related taxa, pairwise comparison is always impaired by the possibility of homoplasious changes. All homoplasy types (parallelisms, convergences, reversals) can lead to similar or even identical sequences in unrelated organisms. Even in the case of sister taxa, pairwise comparison of ITS2 CBCs is illegitimate unless the character state in their last common ancestor is taken into consideration. The discrepancy between a phenetic vs. a phylogenetic approach was highlighted here for two sister species of *Acrochaete *(Figure 4). In one base pair located in the conserved part of Helix 2, *A. viridis *and *A. heteroclada *seem to differ by a single hCBC only (A-U vs. G-U), resulting from pairwise comparison. However, the ancestral state of this pair in their last common ancestor was G-C, and thus, *A. viridis *evolved via CBC (G-C → A-U), whereas its sister species differs from the ancestor by one hCBC (G-C → G-U). Phenetic pairwise comparison would therefore predict possible mating ability, whereas the phylogenetic analysis resolves *A. viridis *as a separate species, likely unable to mate with its sister species.

Our case study in the Ulvales demonstrated several discrepancies in the generally accepted assumptions underlying ITS2 evolution and taxonomic concepts based on ITS2 characters. We hope that this study will stimulate others to investigate ITS2 data in greater detail by directly tracing the evolutionary history of individual characters instead of relying on indirect statistical methods only. As soon as such 'close-up' views on ITS2 evolution are available for other groups of eukaryotes, it may be possible to re-evaluate the significance of ITS2 sequence variations for evolution, taxonomy, and speciation processes in eukaryotes in general.

## Conclusions

The present study of the green algal order Ulvales revealed novel and surprising insights into processes underlying ITS2 evolution and the taxonomic significance of ITS2 characters. **1) Many CBC clades sensu Coleman are paraphyletic**. The CBC clades sensu Coleman are not stable over time, since later evolving CBCs result in new CBC clades which are nested in their 'parent CBC clades' thus changing the status of the former towards paraphyletic grades, here germed CBC grades. **2) The occurrence of CBCs is not restricted to terminal branches and CBC clades are therefore not indicative of recent speciation events**. Instead, mapping of CBCs upon the ITS2 phylogeny reveals spreading of CBCs over both deep and terminal divergences. Most terminal, species-level branches are not associated with CBC events, demonstrating that the genes, which control speciation processes via gametic compatibility evolved considerably faster than the conserved parts of helices 2 and 3 of ITS2. **3) Phenetics can be misleading**. Phenetic comparison of ITS2 base pairs between two taxa can lead to false conclusions when the phylogeny of the organisms is ignored. Therefore, it is essential to map CBCs on the phylogenetic tree in order to determine the evolutionary history of the respective base pair, including homoplasious changes. **4) Hemi-CBCs do not contribute to CBCs**. Throughout the ITS2 phylogeny of the Ulvales, not a single base pair revealed a CBC that represented a two-fold hCBC event of the pathway U-A ⇔ U-G ⇔ C-G, although the individual hCBC events occurred with high frequencies. As a general conclusion, evolutionary divergences characterized by CBCs are mostly not characterized by hCBC, and vice versa. Similarly, ITS2 positions showing CBC-type changes are usually different from base pairs evolving via hCBCs. We conclude that CBCs likely evolved via short-lived non-paired intermediates.

Although the conclusions of this study were derived from ITS2 data of only a single group of algae (Ulvales, Chlorophyta, Viridiplantae), they may well apply to other eukaryotes. Concepts of species delimitation based on presence/absence of CBCs in ITS2 should be applied only after careful analysis of ITS2 evolution and phylogeny.

## Methods

### Cultures, DNA extraction, amplification and sequencing

The investigated strains (taxa in bold in Additional file 7 and Figure 2) were obtained from Sammlung von Algenkulturen, University of Göttingen, Germany (SAG) [119], the Culture Collection of Algae at The University of Texas at Austin (UTEX) [120], the Coimbra Collection of Algae (ACOI) [121], and the Provasoli-Guillard National Center for Culture of Marine Phytoplancton (CCMP) [122]. Two strains from the Culture Collection of Soil Algae at the Institute of Soil Biology, Czech Republic (ISBAL), *Gloeotilopsis paucicellularis *ISBAL 177 and *Gloeotilopsis *sp. ISBAL 1052, have been deposited in the Culture Collection of Algae at the University of Cologne, Germany (CCAC; M3283, M3284) [123] after purification by isolation of zoospores. Cultures were grown in Waris-H medium [124] under the following conditions: temperature: 16°C, photoperiod: 14 hours L/10 hours D, and light intensity: 10 - 30 μmol m^-2 ^s^-1 ^(measured by Light Meter Li-Cor, LI-250A)

Total genomic DNA was extracted using the DNeasy Plant Mini Kit (QIAGEN) and subsequently used for gene amplification by polymerase chain reaction (PCR) and direct sequencing [52], for primers, see Additional file 8. Twelve newly determined ITS2 sequences are available under accession numbers from HE575887 to HE575898 (Additional file 7, taxa in bold).

### Taxon sampling and alignments of ITS2 and 18S rDNA

GenBank database searches and Blast queries revealed about 150 published ITS2 sequences belonging to the order Ulvales. Sequences containing obvious data errors as well as redundant and partial ITS2 sequences were excluded. Finally, 74 published and 12 newly determined ITS2 sequences were subjected to manual alignment, using SeaView 4.1 [125]. The alignment was guided by secondary structures of the ITS2 RNA transcripts (see below).

For the 18S rDNA analyses, 74 sequences were selected as guided by the taxon sampling in the ITS2 alignment. 18S rDNA sequences were aligned manually according to the conserved rRNA secondary structure.

### Consensus ITS2 secondary structure diagram, variability map and nucleotide numbering system

ITS2 secondary structures of all investigated taxa were predicted by comparing RNA folding patterns of complete ITS2 sequences and, if necessary, of single helices, using MFold and RNAstructure. Both methods usually resulted in several alternative foldings for the same ITS2 sequence. The 'true' folding pattern corresponded to the secondary structure model of [4], and was well supported by CBCs and hCBCs, revealed by comparisons among related taxa. To obtain a consensus secondary structure of ITS2 including a variability map, a majority rule consensus sequence at 70% threshold level was calculated via SeaView 4.1 from the ITS2 alignment, and manually displayed as an ITS2 secondary structure diagram (Adobe Illustrator). For each position, the variability category, i.e. the total number of evolutionary changes, was determined by loading sequence data and a ML treefile with PAUP 4.0b10 [126], selecting the Parsimony optimality criterion, and using the 'Describe trees' command with the 'list of changes' option. In addition, expansion segments with length variations across taxa as well as 'non-universal' insertions characterizing only single taxa were specially marked (see Figure 1). 129 'universal' positions, which were unambiguously aligned and present in all Ulvales, were used to introduce an ITS2 nucleotide numbering system (see Results).

### Phylogenetic analyses

Four different methods were performed for phylogenetic analyses: Maximum Likelihood (ML), Distance (Neighbor Joining, NJ), Maximum Parsimony (MP), and Bayesian analyses (MrBayes). The appropriate model of sequence evolution including model parameters was calculated using Akaike Information Criterion (AIC) with ModelTest 3.7 [127], and resulted in GTR+G as the best model for the ITS2 data set and in GTR+I+G for 18S rRNA analyses. These models were used for all analyses in this study except MP. Analyses were calculated by PAUP 4.0b10 (ML, NJ, MP) and MrBayes 3.1.2 [128]. Tree topologies were gained by heuristic searches under the ML criterion, starting with trees obtained by sequential taxon addition or by NJ. 100 ML bootstrap replicates were constrained towards 3000 rearrangements per replicate. MP and NJ bootstrap analyses (1000 replicates) were not constrained.

For Bayesian analyses, two MCMC chains with 2000000 generations were used and 65000 generations were discarded as 'burn in' after estimation with Tracer 1.4 [129]; convergence indicated by a standard deviation between the two MCMC chains below 0.05. Bootstrap values below 50% as well as Bayesian posterior probability below 0.95 were omitted. To determine simple branch lengths (i.e. number of evolutionary steps), we opened ITS2 data and the ML tree of the ITS2 analysis in PAUP, selected the MP criterion (character state optimization: 'DELTRAN'), and displayed the tree by using the 'show branch lengths' option. By excluding all non-paired positions from the alignment, branch lengths referred to double-stranded positions only.

### Mapping of synapomorphic CBCs, hCBCs, and non-compensating substitutions

In order to trace all ITS2 substitutions in the phylogeny of the Ulvales, we applied a modified synapomorphy search. The ITS2 alignment was reduced towards paired (double-stranded) positions, opened with PAUP together with the ML tree file, and screened for synapomorphies as described previously [52,130]. In the resulting 'list of synapomorphies', every character was investigated separately using the 'show reconstructions' option, irrespective of whether it evolved in a homoplasious (e.g. with convergent changes) or non-homoplasious manner. For every change in a given position, the paired position (according to the consensus structure diagram, Figure 1) was screened for presence/absence of a compensatory base change.

## Authors' contributions

LC prepared most new sequences, and prepared the ITS2 alignment, consensus secondary structures, and various ITS2 analyses. BM contributed two new sequences, and prepared the 18S rDNA alignment and analysis. BM and LC wrote the manuscript and together developed ideas and methods to analyze ITS2 data concerning CBC mapping, quantification, and comparison with taxonomic concepts. MM proposed the ITS2 numbering system, provided many ideas concerning evolutionary approaches of CBC/hCBC analyses, and critically read the manuscript. All authors read and approved the final manuscript.

## Competing interests section

The authors declare that they have no competing interests.

## Supplementary Material

Additional file 1 **Selected ITS2 'template' structures of *Ulva *spp. from the ITS2 Database III, showing artificial folding**. All *Ulva *spp. are characterized by (1) the ITS2 Database III identification number, and (2) the accession number of the sequence entry, and (3) the method used for folding in the ITS2 Database III [Method 1 (M1) - direct folding (e.i. derived from e.g. MFold, RNAstructure, Method 2 (M2) - homology modeling]. Click here for file

Additional file 2 **18S rDNA maximum likelihood phylogeny of the Ulvales (74 taxa) based upon 1702 aligned characters**. Habitat preferences as well as presence/absence of scales on zoospores (aplanospores)/gametes are emphasized in the same way as in Figure 2. The branch separating the Capsosiphonaceae, Gomontiaceae and *Pseudoneochloris marina *from the remaining Ulvales was designated as root of the tree. Significances at branches as in Figure 2; bold branches have maximal support by all methods. Note that *Pseudoneochloris marina *diverged as an independent branch, in contrast to the ITS2 phylogeny. Click here for file

Additional file 3 **Evolution of synapomorphic CBCs (Compensatory Base Changes)/hCBCs (hemi-Compensatory Base Changes) in ITS2 of the Ulvales**. Branch lengths (**L **= apomorphic evolutionary changes of the basal branch in Figure 3) referred to the common branch of the clade. Base pairs were labeled by the nucleotide numbering system introduced in Figure 1 (e.g. as **72/108**). Information on **hCBC**s was indicated by [brackets]. 15 H2+3_CBCs (CBCs discovered in the conserved regions of ITS2) were indicated in gray boxes. Unique synapomorphies were flagged as **NHS **(**N**on-**H**omoplasious **S**ynapomorphy), whereas **H**omoplasious **S**ynapomorphies are designated as **HS**. Only **H**omoplasious **S**ynapomorphies were further characterized as (1) parallel CBCs (**PAR)**, (2) parallel hCBCs (**hPAR**), (3) convergent CBCs (**CONV**), (4) reversals of CBCs (**REV**), or (5) reversals of hCBCs (**hREV**). Click here for file

Additional file 4 **List of all substitutions of ITS2 base pairs during the evolution of the Ulvales**. For nucleotide numbers, see Figure 1A. CBCs (blue) and hCBCs (red) were classified into non-homoplasious (NHS) and homoplasious character changes (HS, categorized into parallelisms, convergences, and reversals; or further explanation, see Additional file 3). For every pair, the likely plesiomorphic character status within the Ulvales is given. Moreover, non-compensating base changes are listed here, that involve a pair ⇔ unpair conversion. The conserved regions of helices 2 and 3 were depicted in gray shades. Click here for file

Additional file 5 **Compensatory base changes distributed over conserved regions of helices 2 and 3 of ITS2 in the Ulvales**. All 15 compensatory base changes found in conserved regions of helices 2 and 3 (H2+3_CBCs) were mapped on the consensus secondary structure model of ITS2 in the Ulvales. Comments refer either to their non-homoplasious (NHS) or to homoplasious (HS) status. For further information on universal/non-universal positions see Figure 1. Click here for file

Additional file 6 **Numbers of compensating changes in ITS2 helices diagrammed against branch lengths in the ITS2 phylogeny. A) **The number of CBCs appeared weakly correlated with the length of branches where the CBCs occurred (brown squares with numbers indicating the frequency of CBCs versus evolutionary steps). For branches with > 0 CBCs, the CBC vs. branch length ratio was calculated (CBC_R = 2xCBC/evolutionary steps, blue squares), showing negative correlation with branch lengths. **B) **Hemi-CBCs were not strictly correlated with branch lengths (brown squares with numbers showing the frequency of hCBCs versus evolutionary steps)), but the hCBC vs. branch length ratio (hCBC_R = hCBC/evolutionary steps, blue squares) again clearly showed negative correlation. For both diagrams, branch length calculation was restricted to double-stranded ITS2 positions. Note that the gray colour in the diagrams indicates the area in which no CBCs (**A**) and hCBCs (**B**) occur. Click here for file

Additional file 7 **Strain designations, origins and accession-numbers of nuclear-encoded ITS2 rRNA 86 strains of the Ulvales**. Newly determined sequences are in bold. An asterisk (*) indicates authentic cultures. Click here for file

Additional file 8 **Primers used for PCR amplification/sequencing of ITS2 in the nuclear-encoded rRNA operon of the Ulvales**. Since a few cultures were contaminated with fungi, PCR reactions were performed with specific reverse primers that mismatched with fungal rDNA sequences (labelled 'exFungi'; specific 3'-positions underlined). Click here for file

## References

[B1] Pöll G Braun T Jakovljevic J Neueder A Jakob S Woolford JL Tschochner H Milkereit P rRNA maturation in yeast cells depleted of large ribosomal subunit proteins PLoS One 2009 4 12 e8249 10.1371/journal.pone.0008249 20011513 PMC2788216

[B2] Thomson E Tollervey D The final step in 5.8S rRNA processing is cytoplasmic in *Saccharomyces cerevisiae* Molecular and Cellular Biology 2010 30 4 976 984 10.1128/MCB.01359-09 20008552 PMC2815566

[B3] Zakrzewska-Placzek M Souret FF Sobczyk GJ Green PJ Kufel J *Arabidopsis thaliana *XRN2 is required for primary cleavage in the pre-ribosomal RNA Nucleic Acids Research 2010 38 13 4487 4502 10.1093/nar/gkq172 20338880 PMC2910052

[B4] Mai JC Coleman AW The internal transcribed spacer 2 exhibits a common secondary structure in green algae and flowering plants Journal of Molecular Evolution 1997 44 3 258 271 10.1007/PL00006143 9060392

[B5] Joseph N Krauskopf E Vera MI Michot B Ribosomal internal transcribed spacer 2 (ITS2) exhibits a common core of secondary structure in vertebrates and yeast Nucleic Acids Research 1999 27 23 4533 4540 10.1093/nar/27.23.4533 10556307 PMC148739

[B6] Coleman AW Pan-eukaryote ITS2 homologies revealed by RNA secondary structure Nucleic Acids Research 2007 35 10 3322 3329 10.1093/nar/gkm233 17459886 PMC1904279

[B7] Schultz J Maisel S Gerlach D Müller T Wolf M A common core of secondary structure of the internal transcribed spacer 2 (ITS2) throughout the Eukaryota RNA 2005 11 4 361 364 10.1261/rna.7204505 15769870 PMC1370725

[B8] Geerlings TH Vos JC Raue HA The final step in the formation of 25S rRNA in *Saccharomyces cerevisiae *is performed by 5 '-> 3 ' exonucleases RNA 2000 6 12 1698 1703 10.1017/S1355838200001540 11142370 PMC1370040

[B9] Côté CA Greer CL Peculis BA Dynamic conformational model for the role of ITS2 in pre-rRNA processing in yeast RNA 2002 8 6 786 797 10.1017/S1355838202023063 12088151 PMC1370297

[B10] Fromont-Racine M Senger B Saveanu C Fasiolo F Ribosome assembly in eukaryotes Gene 2003 313 17 42 12957375 10.1016/s0378-1119(03)00629-2

[B11] Babiano R de la Cruz J Ribosomal protein L35 is required for 27SB pre-rRNA processing in *Saccharomyces cerevisiae* Nucleic Acids Research 2010 38 15 5177 5192 10.1093/nar/gkq260 20392820 PMC2926614

[B12] Coleman AW van Oppen MJH Secondary structure of the rRNA ITS2 region reveals key evolutionary patterns in acroporid corals Journal of Molecular Evolution 2008 67 4 389 396 10.1007/s00239-008-9160-y 18781354

[B13] Peyretaillade E Biderre C Peyret P Duffieux F Méténier G Gouy M Michot B Vivaré CP Microsporidian *Encephalitozoon cuniculi*, a unicellular eukaryote with an unusual chromosomal dispersion of ribosomal genes and a LSU rRNA reduced to the universal core Nucleic Acids Research 1998 26 15 3513 3520 10.1093/nar/26.15.3513 9671812 PMC147740

[B14] Gutell RR Larsen L Woese CR Lessons from an evolving rRNA: 16S and 23S rRNA structures from a comparative perspective Microbiological Reviews 1994 58 1 10 26 8177168 10.1128/mr.58.1.10-26.1994 PMC372950

[B15] Coleman AW Comparison of *Eudorina*/*Pleodorina *ITS sequences of isolates from nature with those from experimental hybrids American Journal of Botany 2002 89 9 1523 1530 10.3732/ajb.89.9.1523 21665754

[B16] Coleman AW ITS2 is a double-edged tool for eukaryote evolutionary comparisons Trends in Genetics 2003 19 7 370 375 10.1016/S0168-9525(03)00118-5 12850441

[B17] Coleman AW Vacquier VD Exploring the phylogenetic utility of ITS sequences for animals: A test case for abalone (*Haliotis*) Journal of Molecular Evolution 2002 54 2 246 257 10.1007/s00239-001-0006-0 11821917

[B18] Young I Coleman AW The advantages of the ITS2 region of the nuclear rDNA cistron for analysis of phylogenetic relationships of insects: a *Drosophila *example Molecular Phylogenetics and Evolution 2004 30 1 236 242 10.1016/S1055-7903(03)00178-7 15022773

[B19] Archibald JK Mort ME Crawford DJ Kelly JK Life history affects the evolution of reproductive isolation among species of *Coreopsis *(Asteraceae) Evolution 2005 59 11 2362 2369 16396177

[B20] Coleman AW *Paramecium aurelia *revisited J Eukaryot Microbiol 2005 52 1 68 77 10.1111/j.1550-7408.2005.3327r.x 15702983

[B21] Ahvenniemi P Wolf M Lehtonen MJ Wilson P German-Kinnari M Valkonen JPT Evolutionary diversification indicated by compensatory base changes in ITS2 secondary structures in a complex fungal species, *Rhizoctonia solani* Journal of Molecular Evolution 2009 69 2 150 163 10.1007/s00239-009-9260-3 19609478

[B22] Mullineux T Hausner G Evolution of rDNA ITS1 and ITS2 sequences and RNA secondary structures within members of the fungal genera *Grosmannia *and *Leptographium* Fungal Genetics and Biology 2009 46 11 855 867 10.1016/j.fgb.2009.08.001 19665572

[B23] Kraft LGK Kraft GT Waller RF Investigations into southern Australian *Ulva *(Ulvophyceae, Chlorophyta) taxonomy and molecular phylogeny indicate both cosmopolitanism and endemic cryptic species Journal of Phycology 2010 46 6 1257 1277 10.1111/j.1529-8817.2010.00909.x

[B24] Fabry S Köhler A Coleman AW Intraspecies analysis: comparison of ITS sequence data and gene intron sequence data with breeding data for a worldwide collection of *Gonium pectorale* Journal of Molecular Evolution 1999 48 1 94 101 10.1007/PL00006449 9873081

[B25] Coleman AW The significance of a coincidence between evolutionary landmarks found in mating affinity and a DNA sequence Protist 2000 151 1 1 9 10.1078/1434-4610-00002 10896128

[B26] Coleman AW Is there a molecular key to the level of "biological species" in eukaryotes? A DNA guide Molecular Phylogenetics and Evolution 2009 50 1 197 203 10.1016/j.ympev.2008.10.008 18992828

[B27] Angeler DG Schagerl M Coleman AW Phylogenetic relationships among isolates of *Eudorina *species (Volvocales, Chlorophyta) inferred from molecular and biochemical data Journal of Phycology 1999 35 4 815 823 10.1046/j.1529-8817.1999.3540815.x

[B28] Coleman AW Jaenicke L Starr RC Genetics and sexual behavior of the pheromone producer *Chlamydomonas allensworthii *(Chlorophyceae) Journal of Phycology 2001 37 2 345 349 10.1046/j.1529-8817.2001.037002345.x

[B29] Coleman AW Biogeography and speciation in the *Pandorina/Volvulina *(Chlorophyta) superclade Journal of Phycology 2001 37 5 836 851 10.1046/j.1529-8817.2001.01043.x

[B30] Baldwin BG Kyhos DW Dvorak J Carr GD Chloroplast DNA evidence for a North-American origin of the Hawaiian silversword alliance (Asteraceae) Proceedings of the National Academy of Sciences of the United States of America 1991 88 5 1840 1843 10.1073/pnas.88.5.1840 11607157 PMC51121

[B31] Wu W Zhou RC Huang YL Boufford DE Shi SH Molecular evidence for natural intergeneric hybridization between *Liquidambar *and *Altingia* Journal of Plant Research 2010 123 2 231 239 10.1007/s10265-009-0275-z 19941029

[B32] Amato A Kooistra WHCF Ghiron JHL Mann DG Pröschold T Montresor M Reproductive isolation among sympatric cryptic species in marine diatoms Protist 2007 158 2 193 207 10.1016/j.protis.2006.10.001 17145201

[B33] Casteleyn G Chepurnov VA Leliaert F Mann DG Bates SS Lundholm N Rhodes L Sabbe K Vyverman W *Pseudo-nitzschia pungens *(Bacillariophyceae): A cosmopolitan diatom species? Harmful Algae 2008 7 2 241 257 10.1016/j.hal.2007.08.004

[B34] Pröschold T Bock C Luo W Krienitz L Polyphyletic distribution of bristle formation in Chlorellaceae: *Micractinium*, *Diacanthos*, *Didymogenes *and *Hegewaldia *gen. nov (Trebouxiophyceae, Chlorophyta) Phycological Research 2010 58 1 1 8 10.1111/j.1440-1835.2009.00552.x

[B35] Škaloud P Peksa O Evolutionary inferences based on ITS rDNA and actin sequences reveal extensive diversity of the common lichen alga *Asterochloris *(Trebouxiophyceae, Chlorophyta) Molecular Phylogenetics and Evolution 2010 54 1 36 46 10.1016/j.ympev.2009.09.035 19853051

[B36] MFold http://mfold.rna.albany.edu/?q=mfold/RNA-Folding-Form

[B37] RNAstructure http://rna.urmc.rochester.edu/RNAstructure.html

[B38] Reuter JS Mathews DH RNAstructure: software for RNA secondary structure prediction and analysis BMC Bioinformatics 2010 11 129 10.1186/1471-2105-11-129 20230624 PMC2984261

[B39] 4SALE http://4sale.bioapps.biozentrum.uni-wuerzburg.de/

[B40] Seibel PN Müller T Dandekar T Wolf M Synchronous visual analysis and editing of RNA sequence and secondary structure alignments using 4SALE BMC Research Notes 2008 1 91 10.1186/1756-0500-1-91 18854023 PMC2587473

[B41] ITS2 Database III http://its2.bioapps.biozentrum.uni-wuerzburg.de

[B42] Index Nominum Algarum [INA] http://ucjeps.berkeley.edu/INA.html

[B43] Goertzen LR Cannone JJ Gutell RR Jansen RK ITS secondary structure derived from comparative analysis: implications for sequence alignment and phylogeny of the Asteraceae Molecular Phylogenetics and Evolution 2003 29 2 216 234 10.1016/S1055-7903(03)00094-0 13678678

[B44] Aguilar C Sánchez JA Phylogenetic hypotheses of gorgoniid octocorals according to ITS2 and their predicted RNA secondary structures Molecular Phylogenetics and Evolution 2007 43 3 774 786 10.1016/j.ympev.2006.11.005 17254805

[B45] LaRue B Gaudreau C Bagre HO Charpentier G Generalized structure and evolution of ITS1 and ITS2 rDNA in black flies (Diptera: Simuliidae) Molecular Phylogenetics and Evolution 2009 53 3 749 757 10.1016/j.ympev.2009.07.032 19654048

[B46] Schultz J Wolf M ITS2 sequence-structure analysis in phylogenetics: A how-to manual for molecular systematics Molecular Phylogenetics and Evolution 2009 52 2 520 523 10.1016/j.ympev.2009.01.008 19489124

[B47] Trizzino M Audisio P Antonini G De Biase A Mancini E Comparative analysis of sequences and secondary structures of the rRNA internal transcribed spacer 2 (ITS2) in pollen beetles of the subfamily Meligethinae (Coleoptera, Nitidulidae): potential use of slippage-derived sequences in molecular systematics Molecular Phylogenetics and Evolution 2009 51 2 215 226 10.1016/j.ympev.2008.11.004 19059352

[B48] Keller A Forster F Müller T Dandekar T Schultz J Wolf M Including RNA secondary structures improves accuracy and robustness in reconstruction of phylogenetic trees Biology Direct 2010 5 (1) 10.1186/1745-6150-5-4 PMC2821295 20078867

[B49] Gutell RR Lee JC Cannone JJ The accuracy of ribosomal RNA comparative structure models Current Opinion in Structural Biology 2002 12 3 301 310 10.1016/S0959-440X(02)00339-1 12127448

[B50] Schultz J Müller T Achtziger M Seibel PN Dandekar T Wolf M The internal transcribed spacer 2 database - a web server for (not only) low level phylogenetic analyses Nucleic Acids Research 2006 34 Supplement 2 W704 W707 16845103 10.1093/nar/gkl129 PMC1538906

[B51] Wolf M Achtziger M Schultz J Dandekar T Müller T Homology modeling revealed more than 20,000 rRNA internal transcribed spacer 2 (ITS2) secondary structures RNA 2005 11 11 1616 1623 10.1261/rna.2144205 16244129 PMC1370847

[B52] Marin B Palm A Klingberg M Melkonian M Phylogeny and taxonomic revision of plastid-containing euglenophytes based on SSU rDNA sequence comparisons and synapomorphic signatures in the SSU rRNA secondary structure Protist 2003 154 1 99 145 10.1078/143446103764928521 12812373

[B53] Ruhl MW Wolf M Jenkins TM Compensatory base changes illuminate morphologically difficult taxonomy Molecular Phylogenetics and Evolution 2010 54 2 664 669 10.1016/j.ympev.2009.07.036 19660561

[B54] Fawley MW Fawley KP Hegewald E Taxonomy of *Desmodesmus serratus *(Chlorophyceae, Chlorophyta) and related taxa on the basis of morphological and DNA sequence data Phycologia 2011 50 1 23 56 10.2216/10-16.1

[B55] Krienitz L Bock C Dadheech PK Pröschold T Taxonomic reassessment of the genus *Mychonastes *(Chlorophyceae, Chlorophyta) including the description of eight new species Phycologia 2011 50 1 89 106 10.2216/10-15.1

[B56] Müller T Philippi N Dandekar T Schultz J Wolf M Distinguishing species RNA 2007 13 9 1469 1472 10.1261/rna.617107 17652131 PMC1950759

[B57] Biffin E Harrington MG Crisp MD Craven LA Gadek PA Structural partitioning, paired-sites models and evolution of the ITS transcript in *Syzygium *and Myrtaceae Molecular Phylogenetics and Evolution 2007 43 1 124 139 10.1016/j.ympev.2006.08.013 17070713

[B58] Engelen S Tahi F Predicting RNA secondary structure by the comparative approach: how to select the homologous sequences BMC Bioinformatics 2007 8 464 10.1186/1471-2105-8-464 18045491 PMC2238770

[B59] Rousset F Pélandakis M Solignac M Evolution of compensatory substitutions through G•U intermediate state in *Drosophila *rRNA Proceedings of the National Academy of Sciences of the United States of America 1991 88 22 10032 10036 10.1073/pnas.88.22.10032 1946420 PMC52861

[B60] Tillier ERM Collins RA High apparent rate of simultaneous compensatory base-pair substitutions in ribosomal RNA Genetics 1998 148 4 1993 2002 9560412 10.1093/genetics/148.4.1993 PMC1460107

[B61] Chen Y Carlini DB Baines JF Parsch J Braverman JM Tanda S Stephan W RNA secondary structure and compensatory evolution - Proceedings of Fukuoka International Symposium on Population Genetics Genes & Genetic Systems 1999 74 6 271 286 10.1266/ggs.74.271 10791023

[B62] McCutchan TF Rathore D Li J Compensatory evolution in the human malaria parasite *Plasmodium ovale* Genetics 2004 166 1 637 640 10.1534/genetics.166.1.637 15020451 PMC1470707

[B63] Haag ES Compensatory vs. pseudocompensatory evolution in molecular and developmental interactions Genetica 2007 129 1 45 55 17109184 10.1007/s10709-006-0032-3

[B64] Harrington MG Biffin E Gadek PA Comparative study of the evolution of nuclear ribosomal spacers incorporating secondary structure analyzes within Dodonaeoideae, Hippocastanoideae and Xanthoceroideae (Sapindaceae) Molecular Phylogenetics and Evolution 2009 50 2 364 375 10.1016/j.ympev.2008.11.010 19056501

[B65] Morosyuk SV SantaLucia JJr Cunningham PR Structure and function of the conserved 690 hairpin in *Escherichia coli *16 S ribosomal RNA. III. Functional analysis of the 690 loop Journal of Molecular Biology 2001 307 1 213 228 10.1006/jmbi.2000.4432 11243815

[B66] Varani G McClain WH The G•U wobble base pair EMBO reports 2000 1 1 18 23 10.1093/embo-reports/kvd001 11256617 PMC1083677

[B67] Gautheret D Konings D Gutell RR G•U base pairing motifs in ribosomal RNA RNA 1995 1 8 807 814 7493326 PMC1369321

[B68] Mokdad A Krasovska MV Šponer J Leontis NB Structural and evolutionary classification of G/U wobble basepairs in the ribosome Nucleic Acids Research 2006 34 5 1326 1341 10.1093/nar/gkl025 16522645 PMC1390688

[B69] Gagnon MG Steinberg SV The adenosine wedge: A new structural motif in ribosomal RNA RNA 2010 16 2 375 381 10.1261/rna.1550310 20038632 PMC2811666

[B70] Strazewski P Biala E Gabriel K McClain WH The relationship of thermodynamic stability at a G•U recognition site to tRNA aminoacylation specificity RNA 1999 5 11 490 1494 10.1017/s1355838299991586 PMC1369870 10580477

[B71] Xia T Mathews DH Turner DH Söll DG, Nishimura S, Moore PB Thermodynamics of RNA secondary structure formation Prebiotic chemistry, molecular fossils, nucleosides, and RNA 1999 New York: Elsevier 21 47

[B72] Kern AD Kondrashov FA Mechanisms and convergence of compensatory evolution in mammalian mitochondrial tRNAs Nature Genetics 2004 36 11 1207 1212 10.1038/ng1451 15502829

[B73] Kimura M The role of compensatory neutral mutations in molecular evolution Journal of Genetics 1985 64 1 7 19 10.1007/BF02923549

[B74] Polanco C González AI de la Fuente Á Dover GA Multigene family of ribosomal DNA in *Drosophila melanogaster *reveals contrasting patterns of homogenization for IGS and ITS spacer regions: A possible mechanism to resolve this paradox Genetics 1998 149 1 243 256 9584100 10.1093/genetics/149.1.243 PMC1460117

[B75] Dixon MT Hillis DM Ribosomal RNA secondary structure: compensatory mutations and implications for phylogenetic analysis Molecular Biology and Evolution 1993 10 1 256 267 8450759 10.1093/oxfordjournals.molbev.a039998

[B76] Algaebase http://www.algaebase.org/

[B77] Bown P Plumb J Sánchez-Baracaldo P Hayes P Brodie J Sequence heterogeneity of green (Chlorophyta) endophytic algae associated with a population of *Chondrus crispus *(Gigartinaceae, Rhodophyta) European Journal of Phycology 2003 38 2 153 163 10.1080/0967026031000095525

[B78] Sussmann AV Mable BK DeWreede RE Berbee ML Identification of green algal endophytes as the alternate phase of *Acrosiphonia *(Codiolales, Chlorophyta) using ITS1 and ITS2 ribosomal DNA sequence data Journal of Phycology 1999 35 3 607 614 10.1046/j.1529-8817.1999.3530607.x

[B79] Woolcott GW Iima M King RJ Speciation within *Blidingia minima *(Chlorophyta) in Japan: Evidence from morphology, ontogeny, and analyses of nuclear rDNA its sequence Journal of Phycology 2000 36 1 227 236 10.1046/j.1529-8817.2000.99034.x

[B80] Lindstrom SC Hanic LA Golden L Studies of the green alga *Percursaria dawsonii *(=*Blidingia dawsonii *comb. nov., Kornmanniaceae, Ulvales) in British Columbia Phycological Research 2006 54 1 40 56 10.1111/j.1440-1835.2006.00407.x

[B81] O´Kelly CJ Wysor B Bellows WK *Collinsiella *(Ulvophyceae, Chlorophyta) and other ulotrichalean taxa with shell-boring sporophytes form a monophyletic clade Phycologia 2004 43 1 41 49 10.2216/i0031-8884-43-1-41.1

[B82] Friedl T Evolution of the polyphyletic genus *Pleurastrum *(Chlorophyta): inferences from nuclear-encoded ribosomal DNA sequences and motile cell ultrastructure Phycologia 1996 35 456 469 10.2216/i0031-8884-35-5-456.1

[B83] Blomster J Maggs CA Stanhope MJ Molecular and morphological analysis of *Enteromorpha intestinalis *and *E. compressa *(Chlorophyta) in the British Isles Journal of Phycology 1998 34 2 319 340 10.1046/j.1529-8817.1998.340319.x

[B84] Tan IH Blomster J Hansen G Leskinen E Maggs CA Mann DG Sluiman HJ Stanhope MJ Molecular phylogenetic evidence for a reversible morphogenetic switch controlling the gross morphology of two common genera of green seaweeds, *Ulva *and *Enteromorpha* Molecular Biology and Evolution 1999 16 8 1011 1018 10474897 10.1093/oxfordjournals.molbev.a026190

[B85] Blomster J Bäck S Fewer DP Kiirikki M Lehvo A Maggs CA Stanhope MJ Novel morphology in *Enteromorpha *(Ulvophyceae) forming green tides American Journal of Botany 2002 89 11 1756 1763 10.3732/ajb.89.11.1756 21665602

[B86] Hayden HS Blomster J Maggs CA Silva PC Stanhope MJ Waaland JR Linnaeus was right all along: *Ulva *and *Enteromorpha *are not distinct genera European Journal of Phycology 2003 38 3 277 294 10.1080/1364253031000136321

[B87] Shimada S Hiraoka M Nabata S Iima M Masuda M Molecular phylogenetic analyses of the Japanese *Ulva *and *Enteromorpha *(Ulvales, Ulvophyceae), with special reference to the free-floating *Ulva* Phycological Research 2003 51 2 99 108 10.1111/j.1440-1835.2003.tb00176.x

[B88] Liu F Pang SJ Xu N Shan TF Sun S Hu XA Yang JQ *Ulva *diversity in the Yellow Sea during the large-scale green algal blooms in 2008-2009 Phycological Research 2010 58 4 270 279 10.1111/j.1440-1835.2010.00586.x

[B89] Woolcott GW King RJ *Ulvaria *(Ulvales, Chlorophyta) in eastern Australia: Morphology, anatomy and ontogeny compared with molecular data Botanica Marina 1998 41 1 63 76 10.1515/botm.1998.41.1-6.63

[B90] Lindstrom SC Hanic LA The phylogeny of North American *Urospora *(Ulotrichales, Chlorophyta) based on sequence analysis of nuclear ribosomal genes, introns and spacers Phycologia 2005 44 2 194 201 10.2216/0031-8884(2005)44[194:TPONAU]2.0.CO;2

[B91] Buchheim MA Keller A Koetschan C Förster F Merget B Wolf M Internal transcribed spacer 2 (nu ITS2 rRNA) sequence-structure phylogenetics: towards an automated reconstruction of the green algal tree of life PLoS One 2011 6 2 e16931 10.1371/journal.pone.0016931 21347329 PMC3037400

[B92] Hiraoka M Shimada S Uenosono M Masuda M A new green-tide-forming alga, *Ulva ohnoi *Hiraoka et Shimada sp. nov. (Ulvales, Ulvophyceae) from Japan Phycological Research 2004 51 1 17 29

[B93] Vischer W Über einige kritische Gattungen und die Systematik der Chaetophorales Beihefte zum Botanischen Centralblatt 1933 51 1 100

[B94] Fritsch FE The structure and reproduction of the algae 1956 1 Cambridge, England: Cambridge Univ. Press

[B95] Whitford LA *Heterodictyon planctonicum *L. Whitford and *Chlorosaccus fluidus *Luther: Further notes and corrections Transactions of the American Microscopical Society 1960 79 2 227 229 10.2307/3224089

[B96] Kornmann P Sahling P-H Zur Taxonomie und Entwicklung der *Monostroma*-Arten von Helgoland Helgoland Marine Research 1962 8 3 302 320

[B97] Bliding C A critical survey of European taxa in Ulvales. Part I. *Capsosiphon*, *Percursaria*, *Blidingia*, *Enteromorpha* Opera Botanica 1963 8 3 1 160

[B98] Bliding C A critical survey of European taxa in Ulvales. Part II. *Ulva*, *Ulvaria*, *Monostroma*, *Kornmannia* Botaniska Notiser 1968 121 3 535 629

[B99] Kornmann P Advances in marine phycology on the basis of cultivation Helgoland Marine Research 1970 20 (1 4):39-61

[B100] Kornmann P Codiolophyceae, a new class of Chlorophyta Helgoland Marine Research 1973 25 1 1 13

[B101] Mattox KR Stewart KD Observations on the zoospores of *Pseudendoclonium basiliense *and *Trichosarcina polymorpha *(Chlorophyceae) Canadian Journal of Botany 1973 51 7 1425 1430 10.1139/b73-178

[B102] Moestrup Ø Ultrastructure of the scale-covered zoospores of the green alga *Chaetosphaeridium*, a possible ancestor of the higher plants and bryophytes Biological Journal of the Linnean Society 1974 6 2 111 125 10.1111/j.1095-8312.1974.tb00717.x

[B103] Moestrup Ø On the phylogenetic validity of the flagellar apparatus in green algae and other chlorophyll A and B containing plants Biosystems 1978 10 1-2 117 144 10.1016/0303-2647(78)90035-7 350301

[B104] Swanson JA Floyd GL Fine structure of the zoospores and thallus of *Blidingia minima* Transactions of the American Microscopical Society 1978 97 4 549 558 10.2307/3226170

[B105] Sluiman HJ Roberts KR Stewart KD Mattox KR Comparative cytology and taxonomy of the Ulvaphyceae. I. The zoospore of *Ulothrix zonata *(Chlorophyta) Journal of Phycology 1980 16 4 537 545 10.1111/j.1529-8817.1980.tb03071.x

[B106] Robenek H Melkonian M Comparative ultrastructure of eyespot membranes in gametes and zoospores of the green alga *Ulva lactuca *(Ulvales) Journal of Cell Science 1981 50 1 149 164 7320063 10.1242/jcs.50.1.149

[B107] Hoops HJ Floyd GL Swanson JA Ultrastructure of the biflagellate motile cells of *Ulvaria oxysperma *(Kütz.) Bliding and phylogenetic relationships among ulvaphycean algae American Journal of Botany 1982 69 1 150 159 10.2307/2442841

[B108] Floyd GL O´Kelly CJ Motile cell ultrastructure and the circumscription of the orders Ulotrichales and Ulvales (Ulvophyceae, Chlorophyta) American Journal of Botany 1984 71 1 111 120 10.2307/2443630

[B109] O´Kelly CJ Floyd GL Dube MA The fine structure of motile cells in the genera *Ulvaria *and *Monostroma*, with special reference to the taxonomic position of *Monostroma oxyspermum *(Ulvophyceae, Chlorophyta) Plant Systematics and Evolution 1984 144 3-4 179 199 10.1007/BF00984132

[B110] Watanabe S Floyd GL Ultrastructure of the motile cells of the prostrate filamentous green algae *Protoderma sarcinoidea *and *Chamaetrichon capsulatum* Plant Systematics and Evolution 1992 179 1-2 73 87 10.1007/BF00938020

[B111] Leonardi PI Correa JA Cáceres EJ Ultrastructure and taxonomy of the genus *Endophyton *(Ulvales, Ulvophyceae) European Journal of Phycology 1997 32 2 175 183

[B112] Nakayama T Inouye I Ultrastructure of the biflagellate gametes of *Collinsiella cava *(Ulvophyceae, Chlorophyta) Phycological Research 2000 48 2 63 73 10.1111/j.1440-1835.2000.tb00198.x

[B113] Watanabe S Kuroda N Maiwa F Phylogenetic status of *Helicodictyon planctonicum *and *Desmochloris halophila *gen. et comb. nov. and the definition of the class Ulvophyceae (Chlorophyta) Phycologia 2001 40 5 421 434 10.2216/i0031-8884-40-5-421.1

[B114] Hoef-Emden K Revision of the genus *Cryptomonas *(Cryptophyceae) II: incongruences between the classical morphospecies concept and molecular phylogeny in smaller pyrenoid-less cells Phycologia 2007 46 4 402 428 10.2216/06-83.1

[B115] Krüger D Gargas A Secondary structure of ITS2 rRNA provides taxonomic characters for systematic studies - a case in Lycoperdaceae (Basidiomycota) Mycological Research 2008 112 3 316 330 10.1016/j.mycres.2007.10.019 18342242

[B116] Miller TL Adlard RD Bray RA Justine J-L Cribb TH Cryptic species of *Euryakaina *n. g. (Digenea: Cryptogonimidae) from sympatric lutjanids in the Indo-West Pacific Systematic parasitology 2010 77 3 185 204 10.1007/s11230-010-9266-7 20960090

[B117] Schmitt S Hentschel U Zea S Dandekar T Wolf M ITS-2 and 18S rRNA gene phylogeny of Aplysinidae (Verongida, Demospongiae) Journal of Molecular Evolution 2005 60 3 327 336 10.1007/s00239-004-0162-0 15871043

[B118] Wiemers M Keller A Wolf M ITS2 secondary structure improves phylogeny estimation in a radiation of blue butterflies of the subgenus *Agrodiaetus* (Lepidoptera: Lycaenidae: *Polyommatus*). BMC Evolutionary Biology 2009 9 300 10.1186/1471-2148-9-300 20035628 PMC2810301

[B119] Sammlung von Algenkulturen, University of Göttingen, Germany (SAG) http://sagdb.uni-goettingen.de/

[B120] Culture Collection of Algae at The University of Texas at Austin (UTEX) http://web.biosci.utexas.edu/utex/

[B121] Coimbra Collection of Algae (ACOI) http://acoi.ci.uc.pt/

[B122] Provasoli-Guillard National Center for Culture of Marine Phytoplancton (CCMP) https://ccmp.bigelow.org/

[B123] Culture Collection of Algae at the University of Cologne, Germany (CCAC) http://www.ccac.uni-koeln.de/

[B124] McFadden GI Melkonian M Use of Hepes buffer for microalgal culture media and fixation for electron microscopy Phycologia 1986 25 4 551 557 10.2216/i0031-8884-25-4-551.1

[B125] SeaView 4.1 http://pbil.univ-lyon1.fr/software/seaview.html

[B126] Swofford DL PAUP*. Phylogenetic Analysis Using Parsimony (*and Other Methods). Version 4 2000 Sunderland, Massachusetts: Sinauer Associates

[B127] Posada D jModelTest: phylogenetic model averaging Molecular Biology and Evolution 2008 25 7 1253 1256 10.1093/molbev/msn083 18397919

[B128] Ronquist F Huelsenbeck JP MrBayes 3: Bayesian phylogenetic inference under mixed models Bioinformatics 2003 19 12 1572 1574 10.1093/bioinformatics/btg180 12912839

[B129] Tracer 1.4 http://tree.bio.ed.ac.uk/software/tracer/

[B130] Marin B Nowack ECM Melkonian M A plastid in the making: Evidence for a second primary endosymbiosis Protist 2005 156 4 425 432 10.1016/j.protis.2005.09.001 16310747

