# Supplementary material for: A close-up view on ITS2 evolution and speciation - a case study in the Ulvophyceae (Chlorophyta, Viridiplantae)
Source: BMC Evol Biol. 2011 Sep 20;11:262. doi: 10.1186/1471-2148-11-262 (PMC3225284; doi:10.1186/1471-2148-11-262)
Supplement: Additional file 1 — Selected ITS2 'template' structures of Ulva spp. from the ITS2 Database III, showing artificial folding. All Ulva spp. are characterized by (1) the ITS2 Database III identification number, and (2) the accession number of the sequence entry, and (3) the method used for folding in the ITS2 Database III [Method 1 (M1) - direct folding (e.i. derived from e.g. MFold, RNAstructure, Method 2 (M2) - homology modeling]. [file 1471-2148-11-262-S1.PDF]

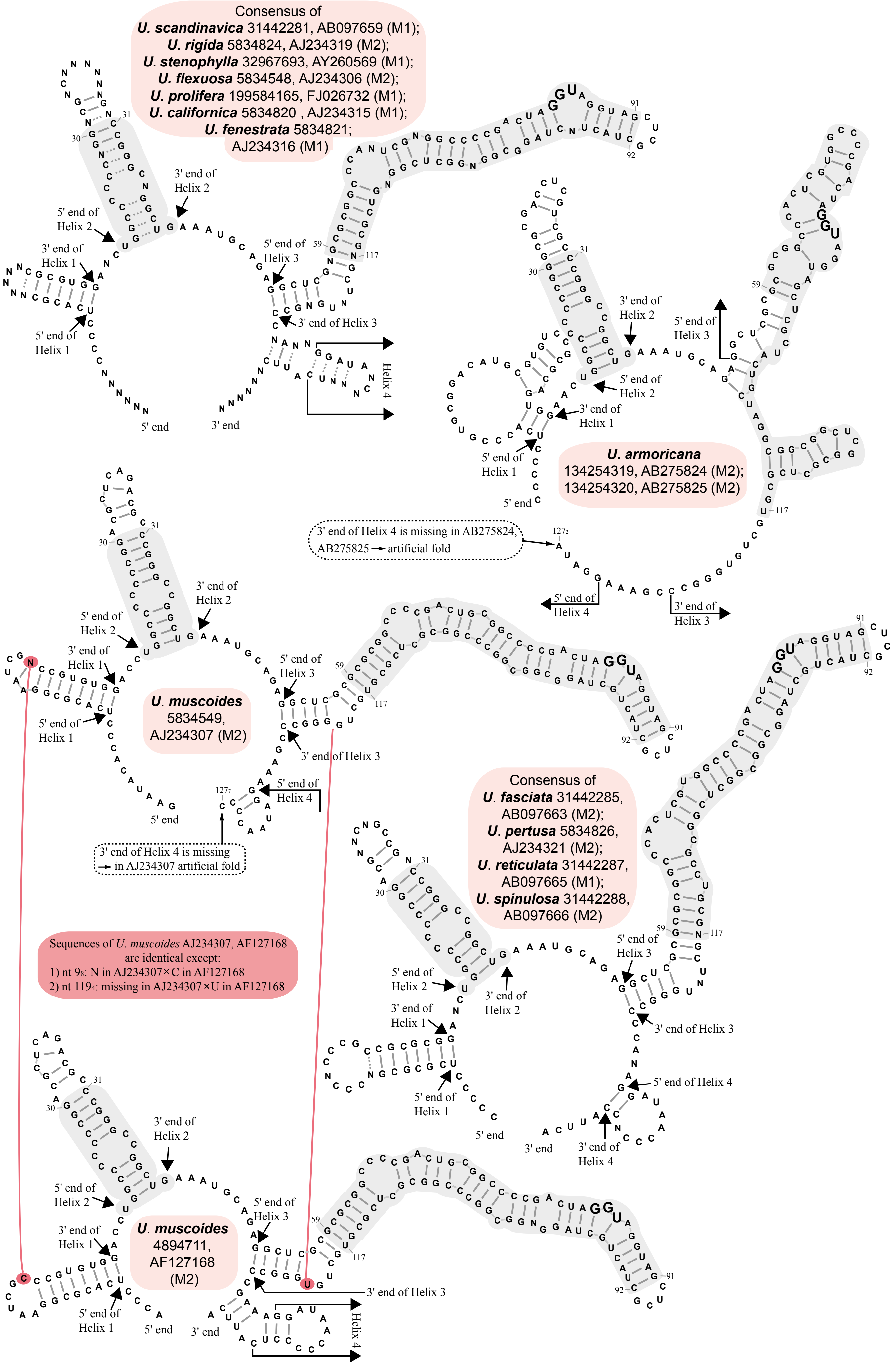

Consensus of
U. scandinavica 31442281, AB097659 (M1);
U. rigida 5834824, AJ234319 (M2);
U. stenophylla 32967693, AY260569 (M1);
U. flexuosa 5834548, AJ234306 (M2);
U. prolifera 199584165, FJ026732 (M1);
U. californica 5834820 , AJ234315 (M1);
U. fenestrata 5834821;
AJ234316 (M1)
5' end of Helix 1
3' end of Helix 1
5' end of Helix 2
3' end of Helix 2
5' end of Helix 3
3' end of Helix 3
Helix 4
5' end
3' end
U. armoricana
134254319, AB275824 (M2);
134254320, AB275825 (M2)
3' end of Helix 4 is missing in AB275824, AB275825 → artificial fold
5' end of Helix 4
U. muscoides
5834549,
AJ234307 (M2)
3' end of Helix 4 is missing → in AJ234307 artificial fold
Sequences of U. muscoides AJ234307, AF127168 are identical except:
1) nt 98: N in AJ234307×C in AF127168
2) nt 1194: missing in AJ234307×U in AF127168
Consensus of
U. fasciata 31442285, AB097663 (M2);
U. pertusa 5834826, AJ234321 (M2);
U. reticulata 31442287, AB097665 (M1);
U. spinulosa 31442288, AB097666 (M2)
3' end of Helix 4
U. muscoides
4894711,
AF127168
(M2)
