# Supplementary figures and images for: A close-up view on ITS2 evolution and speciation - a case study in the Ulvophyceae (Chlorophyta, Viridiplantae)
Source: BMC Evol Biol. 2011 Sep 20;11:262. doi: 10.1186/1471-2148-11-262 (PMC3225284; doi:10.1186/1471-2148-11-262)

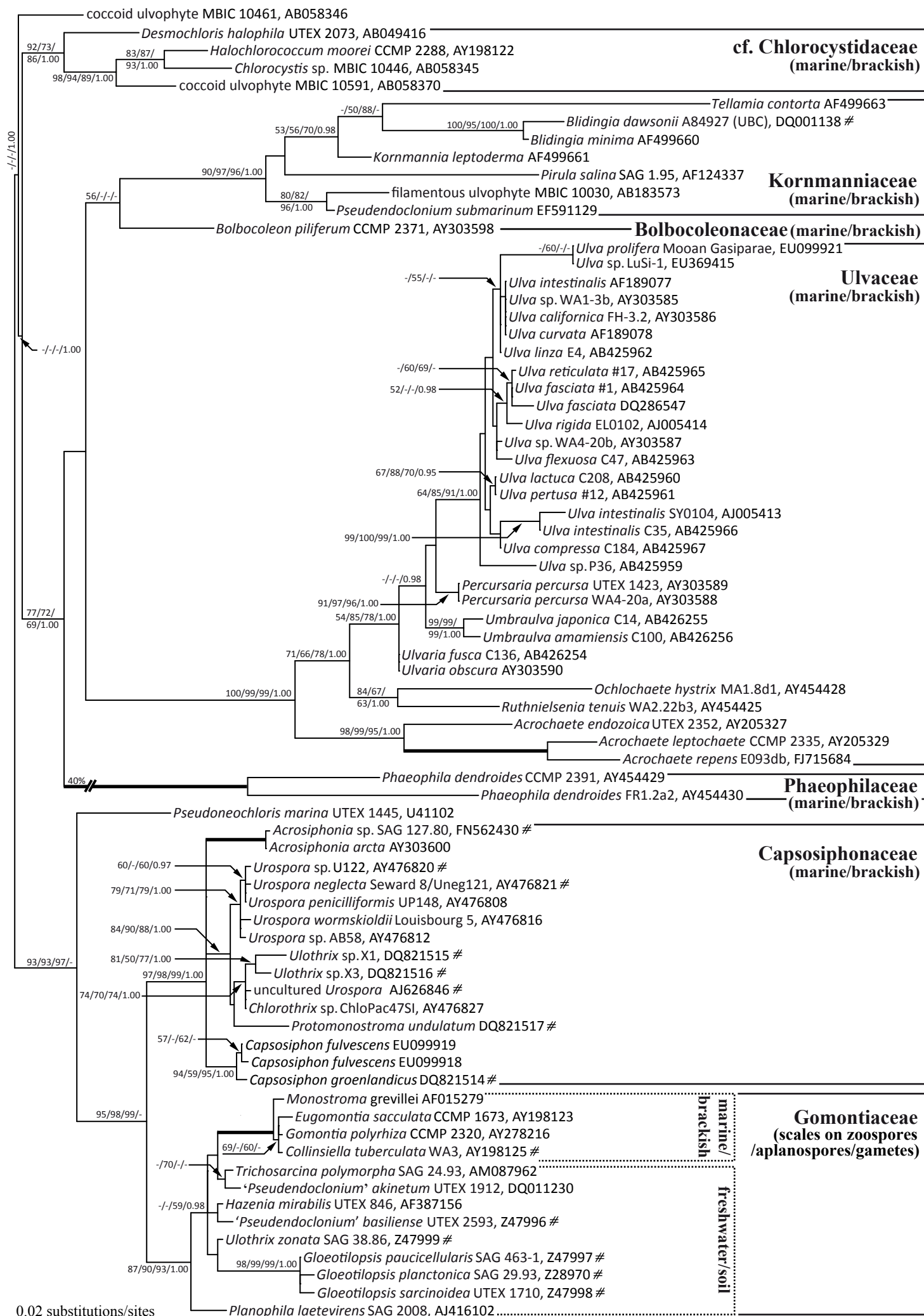

Supplement: Additional file 2 — 18S rDNA maximum likelihood phylogeny of the Ulvales (74 taxa) based upon 1702 aligned characters. Habitat preferences as well as presence/absence of scales on zoospores (aplanospores)/gametes are emphasized in the same way as in Figure 2. The branch separating the Capsosiphonaceae, Gomontiaceae and Pseudoneochloris marina from the remaining Ulvales was designated as root of the tree. Significances at branches as in Figure 2; bold branches have maximal support by all methods. Note that Pseudoneochloris marina diverged as an independent branch, in contrast to the ITS2 phylogeny. [file 1471-2148-11-262-S2.PDF]

A)

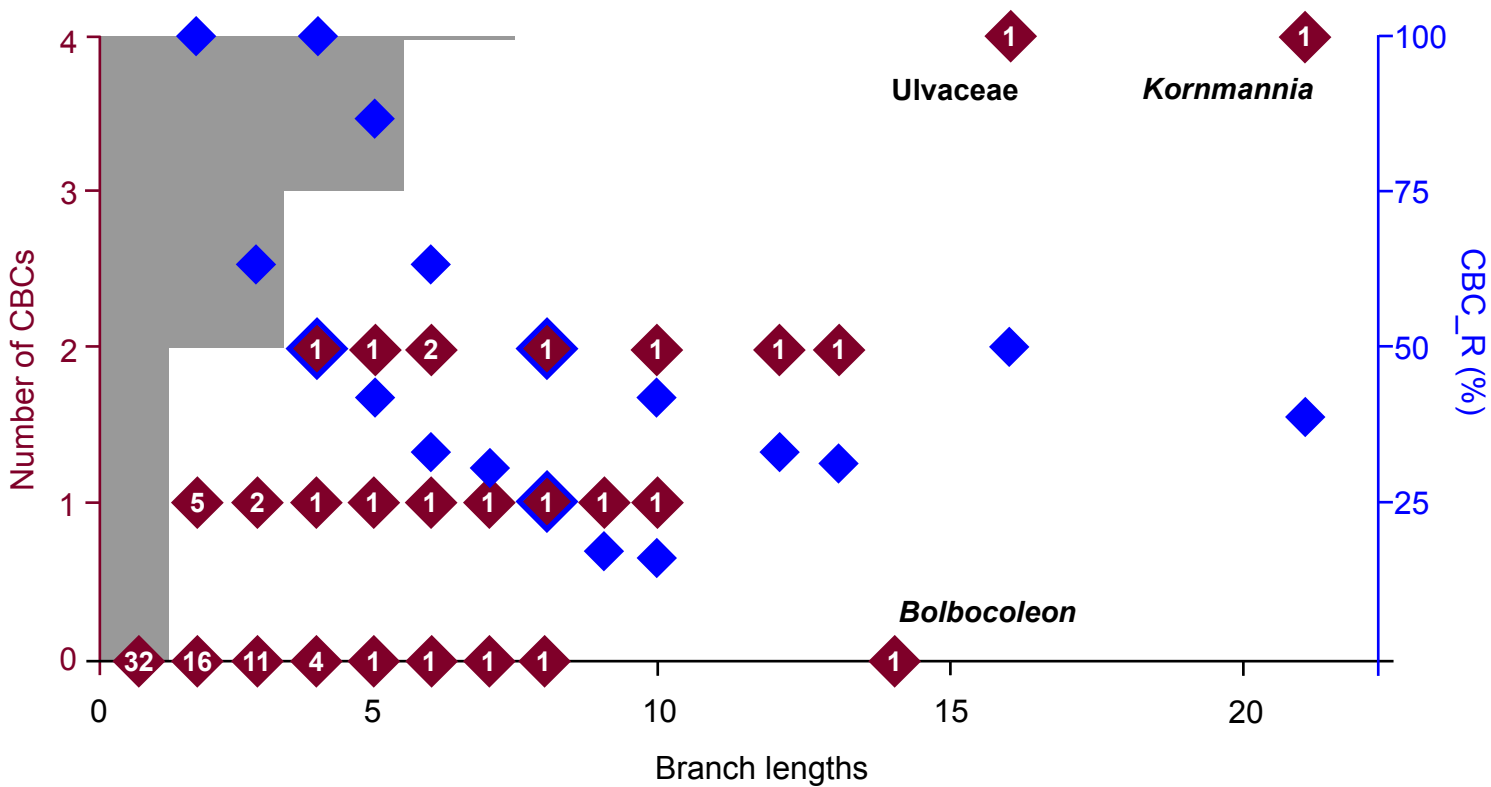

B)

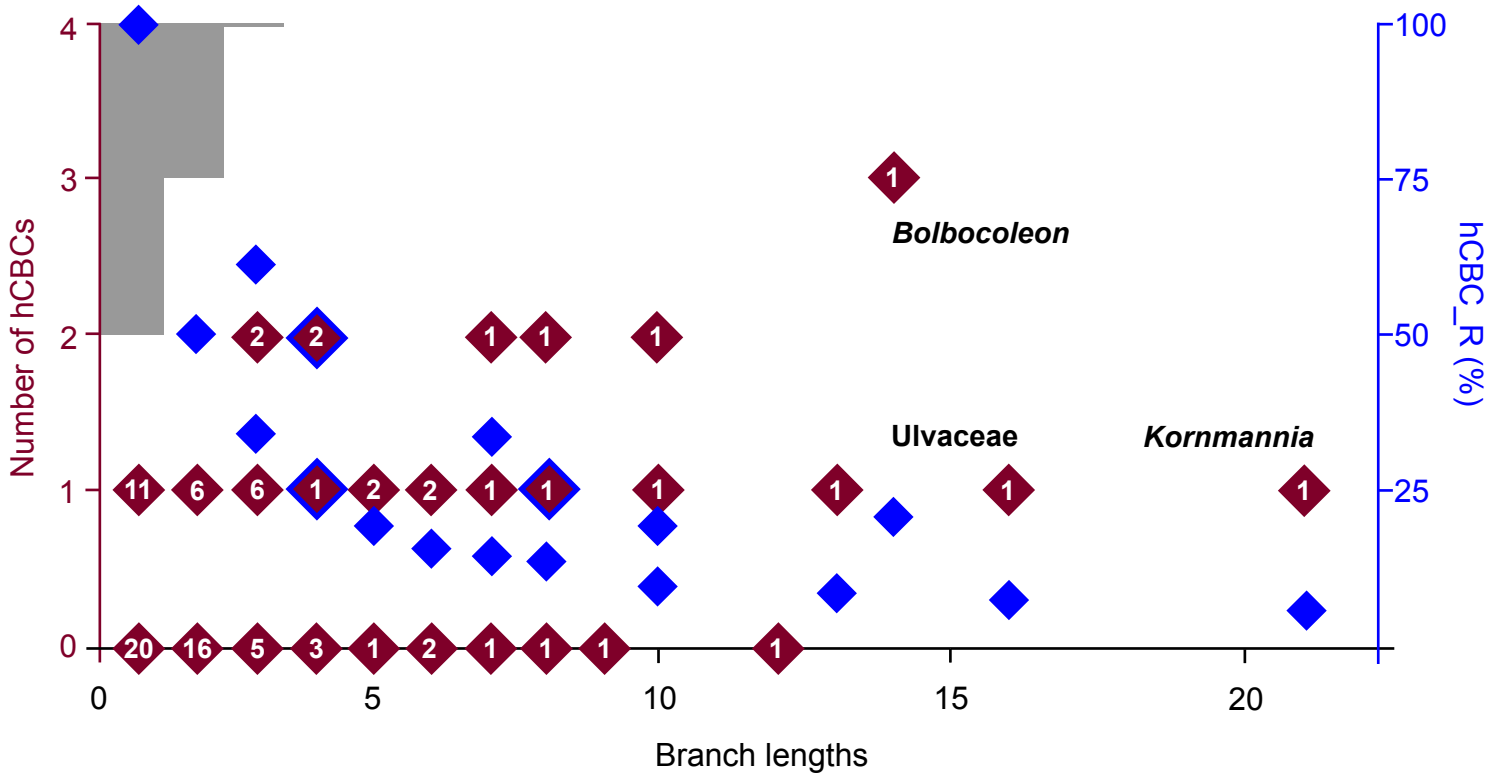

Supplement: Additional file 6 — Numbers of compensating changes in ITS2 helices diagrammed against branch lengths in the ITS2 phylogeny. A) The number of CBCs appeared weakly correlated with the length of branches where the CBCs occurred (brown squares with numbers indicating the frequency of CBCs versus evolutionary steps). For branches with > 0 CBCs, the CBC vs. branch length ratio was calculated (CBC_R = 2xCBC/evolutionary steps, blue squares), showing negative correlation with branch lengths. B) Hemi-CBCs were not strictly correlated with branch lengths (brown squares with numbers showing the frequency of hCBCs versus evolutionary steps)), but the hCBC vs. branch length ratio (hCBC_R = hCBC/evolutionary steps, blue squares) again clearly showed negative correlation. For both diagrams, branch length calculation was restricted to double-stranded ITS2 positions. Note that the gray colour in the diagrams indicates the area in which no CBCs (A) and hCBCs (B) occur. [file 1471-2148-11-262-S6.PDF]
