# Supplementary material for: A close-up view on ITS2 evolution and speciation - a case study in the Ulvophyceae (Chlorophyta, Viridiplantae)
Source: BMC Evol Biol. 2011 Sep 20;11:262. doi: 10.1186/1471-2148-11-262 (PMC3225284; doi:10.1186/1471-2148-11-262)
Supplement: Additional file 3 — Evolution of synapomorphic CBCs (Compensatory Base Changes)/hCBCs (hemi-Compensatory Base Changes) in ITS2 of the Ulvales. Branch lengths (L = apomorphic evolutionary changes of the basal branch in Figure 3) referred to the common branch of the clade. Base pairs were labeled by the nucleotide numbering system introduced in Figure 1 (e.g. as 72/108). Information on hCBCs was indicated by [brackets]. 15 H2+3_CBCs (CBCs discovered in the conserved regions of ITS2) were indicated in gray boxes. Unique synapomorphies were flagged as NHS (Non-Homoplasious Synapomorphy), whereas Homoplasious Synapomorphies are designated as HS. Only Homoplasious Synapomorphies were further characterized as (1) parallel CBCs (PAR), (2) parallel hCBCs (hPAR), (3) convergent CBCs (CONV), (4) reversals of CBCs (REV), or (5) reversals of hCBCs (hREV). [file 1471-2148-11-262-S3.PDF]

Clade	L	Nucleotide positions of CBC and [hCBC]/Evolutionary change NHS	HS	Characterization of HS CBCs and [hCBCs]
Kornmanniaceae + Bolbocoleonaceae	10	**28/33**: C-G => **G-C**	**54/121**: U-A => **A-U**	**54/121**: U-A => **A-U** parallel in *Collinsiella* = **PAR 5**, and **A-U** convergent in *Gloeotilopsis sarcinoidea* UTEX 1710 (U-G =>A-U) = **CONV 1**
			[**126/128**: G-C => G-**U**]	[**126/128**: G-C => G-**U**] parallel in Gomontiaceae (marine/ brackish) + *Ulothrix zonata*, *Acrochaete* + '*Umbraulva japonica*', *Ulva muscoides* and *Ulva lactuca* clade = **hPAR 14**
Kornmanniaceae	6	----	**55/120**: U-A => **G-C**	**55/120**: U-A => **G-C** parallel in '*Blidingia minima*' = **PAR 6**
			[**62/114**: U-G => **C**-G]	[**62/114**: U-G => **C**-G] parallel in Ulvaceae = **hPAR 10**
			**78/102**: U-A => **G-C**	**78/102**: U-A => **G-C** parallel in Ulvaceae = **PAR 7**
Kornmannia	21	**23/38**: C-G => **U-A**	**55/120**: G-C => **A-U**	**55/120**: G-C => **A-U**, **A-U** convergent in *Acrosiphonia* (U-A => A-U) = **CONV 3**
		**67/110**: C-G => **U-A**	[**72/108**: G-C => G-**U**]	[**72/108**: G-C => G-**U**] parallel in Gomontiaceae + Capsosiphonaceae and *Ulva* = **hPAR 12**
		**79/101**: G-C => **A-U**		
Blidingia chadefaudii	2	[**60/116**: C-G **=>** **U**-G]	----	----
Bolbocoleonaceae	14	[**68/109**: U-G **=>** **C**-G]	[**20/41**: G-U **=>** G-**C**]	[**20/41**: G-U => G-**C**] parallel in *Gloeotilopsis* clade = **hPAR 1**

			[**21/40**: G-C **=>** G-**U**]	[**21/40**: G-C => G-**U**] parallel in *Acrochaete heteroclada* = **hPAR 2**
Ulvaceae	16	**29/32**: A-U => **G-C**<br>**54/121**: U-A => **C-G**<br>**75/105**: A-U => **C-G**	[**62/114**: U-G => **C**-G]<br>**78/102**: A-U => **G-C**	[**62/114**: U-G => **C**-G] parallel in Kornmanniaceae = **hPAR 10**<br>**78/102**: U-A => **G-C** parallel in Kornmanniaceae = **PAR 7**
Ulva + *Percursaria* + *Ulvaria*	3	----	[**55/120**: U-A **=>** U-**G**]	[**55/120**: U-A => U-**G**] parallel in *Monostroma* excluding *M. grevillei* AJ000205 and *Gloeotilopsis sarcinoidea* UTEX 1710 = **hPAR 6**
Ulva	10	**8/11**: C-G => **G-C**	----<br>[**58/118**: C-G => **U**-G]<br>[**72/108**: G-C => G-**U**]	[**58/118**: C-G => **U**-G] parallel in *Ulva californica* AB280867 = **hPAR 7**<br>[**72/108**: G-C => G-**U**] parallel in Gomontiaceae + Capsosiphonaceae and *Kornmannia* = **hPAR 12**
Ulva flexuosa + *Ulva californica* AJ234315	1	----	[**59/117**: G-C => G-**U**]	[**59/117**: G-C => G-**U**] parallel in *Acrochaete* sp. EF595429, *Ulva tanneri* and *Ulva linza* AJ012276, AJ000203 + *U. prolifera* + *U. lactuca* clade + *U. muscoides* + *U. taeniata* + *U. reticulata* clade + *U. californica* AB280867 + *Ulva* sp. EU933983 = **hPAR 8**
Ulva californica AJ234315	2	----	[**55/120**: U-G => U-**A**]	[**55/120**: U-G => U-**A**] reversal in *Ulva* + *Percursaria* + *Ulvaria* (U-A => U-G) = **hREV 1**
Ulva linza AJ012276,	1	----	[**59/117**: G-C => G-**U**]	[**59/117**: G-C => G-**U**] parallel in *Acrochaete* sp. EF595429,

AJ000203 + *U. prolifera* + *U. lactuca* clade + *U. muscoides* + *U. taeniata* + *U. reticulata* clade + *U. californica* AB280867 + *Ulva* sp. EU933983				*Ulva tanneri* and *Ulva flexuosa* + *Ulva californica* AJ234315 = **hPAR 8**
Ulva lactuca clade + *U. muscoides* + *U. taeniata* + *U. reticulata* clade + *U. californica* AB280867 + *Ulva* sp. EU933983	2	----	[**58/118**: U-G => **C**-G]	[**58/118**: U-G => **C**-G] reversal in *Ulva* (C-G => U-G)= **hREV 2**
Ulva lactuca clade	3	----	[**126/128**: G-C => G-**U**]	[**126/128**: G-C => G-**U**] parallel in Kornmanniaceae + Bolbocoleonaceae, Gomontiaceae (marine/ brackish) + *Ulothrix zonata*, *Acrochaete* + '*Umbraulva japonica*' and *Ulva muscoides* = **hPAR 14**
Ulva muscoides + *Ulva taeniata*	2	----	[**72/108**: G-U => G-**C**]	[**72/108**: G-U => G-**C**] reversal in *Ulva* (G-C => G-U) = **hREV 6**
Ulva muscoides	3	[**8/11**: G-C => G-**U**]	[**126/128**: G-C => G-**U**]	[**126/128**: G-C => G-**U**] parallel in Kornmanniaceae + Bolbocoleonaceae, Gomontiaceae (marine/ brackish) + *Ulothrix zonata*, *Acrochaete* + '*Umbraulva japonica*' and *Ulva lactuca* clade = **hPAR 14**

Ulva taeniata	3	----	[**59/117**: G-U => G-**C**]	[**59/117**: G-U => G-**C**] parallel in *Ulva fasciata* + *Ulva pertusa* = **hPAR 9**, and reversal in *Ulva linza* AJ012276, AJ000203 + *U. prolifera* + *U. lactuca* clade + *U. muscoides* + *U. taeniata* + *U. reticulata* clade + *U. californica* AB280867 + *Ulva* sp. EU933983 (<u>G-C</u> => G-U) = **hREV 5**
Ulva reticulata clade	4	**6/13**: A-U => **G-C**	[**63/113**: G-C => G-**U**]	[**63/113**: G-C => G-**U**] parallel in Ulvales sp. EF595507 + *Acrochaete* sp. EF595429 = **hPAR 11**
Ulva fasciata + *Ulva pertusa*	1	----	[**59/117**: G-U => G-**C**]	[**59/117**: G-U => G-**C**] parallel in *Ulva taeniata* = **hPAR 9**, and reversal in *Ulva linza* AJ012276, AJ000203 + *U. prolifera* + *U. lactuca* clade + *U. muscoides* + *U. taeniata* + *U. reticulata* clade + *U. californica* AB280867 + *Ulva* sp. EU933983 (<u>G-C</u> => G-U) = **hREV 4**
Ulva californica AB280867	2	----	[**58/118**: C-G => **U**-G]	[**58/118**: C-G => **U**-G] parallel in *Ulva* = **hPAR 7**, and reversal in *Ulva lactuca* clade + *U. muscoides* + *U. taeniata* + *U. reticulata* clade + *U. californica* AB280867 + *Ulva* sp. EU933983 (<u>U-G</u> => C-G) = **hREV 3**
Ulva tanneri	3	----	[**27/34**: C-G => **U**-G]<br><br>[**59/117**: G-C => G-**U**]	[**27/34**: C-G => **U**-G] parallel in Capsosiphonaceae and *Monostroma nitidum* + *M. arcticum* = **hPAR 4**<br><br>[**59/117**: G-C => G-**U**] parallel in *Acrochaete* sp. EF595429, *Ulva* flexuosa + *U. californica* AJ234315 and *Ulva linza* AJ012276, AJ000203 + *U. prolifera* + *U. lactuca* clade + *U.*

				muscoides + *U. taeniata* + *U. reticulata* clade + *U. californica* AB280867 + *Ulva* sp. EU933983 = **hPAR 8**
Percursaria	7	[**125/129**: G-C => G-**U**]	**8/11**: C-G => **U-A**	**8/11**: C-G => **U-A** parallel in Gomontiaceae = **PAR 2**
Acrochaete + '*Umbraulva japonica*'	8	[**80/100**: C-G **=>** **U**-G]	[**126/128**: G-C **=>** G-**U**]	[**126/128**: G-C => G-**U**] parallel in Kornmanniaceae + Bolbocoleonaceae, Gomontiaceae (marine/ brackish) + *Ulothrix zonata*, *Ulva muscoides* and *Ulva lactuca* clade = **hPAR 14**
Acrochaete viridis	3	**21/40**: G-C => **A-U**	----	----
Acrochaete heteroclada	8	**74/106**: C-G => **U-A**	[**21/40**: G-C => G-**U**]	[**21/40**: G-C => G-**U**] parallel in Bolbocoleonaceae = **hPAR 2**
Acrochaete repens	2	[**73/107**: G-C => G-**U**]	----	----
Acrochaete sp. EF595429	4	----	[**59/117**: G-C => G-**U**]	[**59/117**: G-C => G-**U**] parallel in *Ulva tanneri*, *U.* flexuosa + *U. californica* AJ234315 and *Ulva linza* AJ012276, AJ000203 + *U. prolifera* + *U. lactuca* clade + *U. muscoides* + *U. taeniata* + *U. reticulata* clade + *U. californica* AB280867 + *Ulva* sp. EU933983 = **hPAR 8**
			[**63/113**: G-C => G-**U**]	[**63/113**: G-C => G-**U**] parallel in Ulvales sp. EF595507, *Ulva reticulata* clade = **hPAR 11**
'*Umbraulva japonica*', *Acrochaete* sp. EF595429, *Acrochaete* sp. EF595413, *Acrochaete* sp. EF595372	7	[**78/102**: G-C => G-**U**]	----	----

		[**90/93**: A-U => **G**-U]		
Capsosiphonaceae + Gomontiaceae	6	----	[**72/108**: G-C => G-**U**]	[**72/108**: G-C => G-**U**] parallel in *Kornmannia* and *Ulva* = **hPAR 12**
Capsosiphonaceae	1	----	[**27/34**: C-G => **U**-G]	[**27/34**: C-G => **U**-G] parallel in *Ulva tanneri* and *Monostroma arcticum* + *M. nitidum* = **hPAR 4**
Capsosiphonaceae excluding *Protomonostroma undulatum*	1	----	[**22/39**: C-G => **U**-G]	[**22/39**: C-G => **U**-G] parallel in *Monostroma* and *Gloeotilopsis* clade excluding *Gloeotilopsis* sp. M3284 = **hPAR 3**
Acrosiphonia	6	----	**30/31**: G-C => **C-G**<br>**55/120**: U-A => **A-U**	**30/31**: G-C => **C-G** parallel in Gomontiaceae excluding *Chamaetrichon capsulatum* and '*Pseudendoclonium basiliense*' = **PAR 4**<br>**55/120**: U-A => **A-U** convergent in *Kornmannia* (G-C => A-U) = **CONV 3**
Pseudoneochloris marina	12	----	**7/12**: U-A => **C-G**<br>**8/11**: U-A => **C-G**	**7/12**: U-A => **C-G** parallel in *Monostroma* and *Gloeotilopsis* sp. ACOI + *Gloeotilopsis sarcinoidea* UTEX 1710 = **PAR 1**<br>**8/11**: U-A => **C-G** parallel in *Gloeotilopsis paucicellularis Gloeotilopsis* sp. M3284 = **PAR 3**
Urospora sp. AY476812 + *Urospora wormskioldii*	1	----	[**91/92**: G-C => G-**U**]	[**91/92**: G-C => G-**U**] parallel in Gomontiaceae (marine/ brackish) = **hPAR 13**
Ulvales sp. EF595507 +	1	[**8/11**: U-A => U-**G**]	----	----

Ulvales sp. EF595508 + Ulvales sp. EF595509				
Ulvales sp. EF595507	1	----	[**63/113**: G-C => G-**U**]	[**63/113**: G-C => G-**U**] parallel in *Acrochaete* sp. EF595429 + *Ulva reticulata* clade = **hPAR 11**
'*Blidingia minima*'	9	----	**55/120**: U-A => **G-C**	**55/120**: U-A => **G-C** parallel in Kornmanniaceae = **PAR 6**
'*Blidingia minima*' AJ000206	3	----	[**54/121**: U-A => U-**G**]	[**54/121**: U-A => U-**G**] parallel in *Gloeotilopsis* clade = **hPAR 5**
'*Blidingia minima*' EF595512	6	**9/10**: C-G => **G-C**	----	----
Gomontiaceae	2	----	**8/11**: C-G => **U-A**	**8/11**: C-G => **U-A** parallel in *Percursaria* = **PAR 2**
Gomontiaceae excluding *Chamaetrichon capsulatum* and '*Pseudendoclonium basiliense*'	2	----	**30/31**: G-C => **C-G**	**30/31**: G-C => **C-G** parallel in *Acrosiphonia*= **PAR 4**
Gomontiaceae (marine/ brackish) + *Ulothrix zonata*	3	**53/122**: G-C => **A-U**	[**126/128**: G-C **=>** G-**U**]	[**126/128**: G-C => G-**U**] parallel in Kornmanniaceae + Bolbocoleonaceae, *Acrochaete* + '*Umbraulva japonica*', *Ulva muscoides* and *Ulva lactuca* clade = **hPAR 14**
Gomontiaceae (marine/ brackish)	13	**90/93**: A-U => **G-C** **125/129**: G-C => **A-U**	[**91/92**: G-C => G-**U**]	[**91/92**: G-C => G-**U**] parallel in *Urospora* sp. AY476812 + *Urospora wormskioldii* = **hPAR 13**
Monostroma	5	**30/31**: C-G => **U-A**	**7/12**: U-A => **C-G**	**7/12**: U-A => **C-G** parallel in *Pseudoneochloris marina* and *Gloeotilopsis* sp. ACOI + *G. sarcinoidea* UTEX 1710 = **PAR 1**

			[**22/39**: C-G => **U**-G]	[**22/39**: C-G => **U**-G] parallel in Capsosiphonaceae excluding *Protomonostroma undulatum*, *Gloeotilopsis* clade excluding Gloeotilopsis sp. M3284 = **hPAR 3**
Monostroma excluding *M. grevillei* AJ000205	1	----	[**55/120**: U-A => U-**G**]	[**55/120**: U-A => U-**G**] parallel in *Ulva* + *Percursaria* + *Ulvaria*, *Gloeotilopsis sarcinoidea* UTEX 1710 = **hPAR 6**
Monostroma arcticum + *M. nitidum*	1	----	[**27/34**: C-G => **U**-G]	[**27/34**: C-G => **U**-G] parallel in *Ulva tanneri* and Capsosiphonaceae = **hPAR 4**
Collinsiella	8	**23/38**: C-G => **G-C**	**54/121**: U-A => **A-U**	**54/121**: U-A => **A-U** parallel in Kornmanniaceae + Bolboco-leonaceae = **PAR 5**, and **A-U** convergent in *Gloeotilopsis sarcinoidea* UTEX 1710 (U-G=>A-U) = **CONV 2**
Gloeotilopsis clade	4	----	[**20/41**: G-U => G-**C**]<br>[**54/121**: U-A => U-**G**]	[**20/41**: G-U => G-**C**] parallel in Bolbocoleonaceae = **hPAR 1**<br>[**54/121**: U-A => U-**G**] parallel in *Blidingia minima* AJ000206 = **hPAR 5**
Gloeotilopsis clade excluding *Gloeotilopsis* sp. M3284	1	----	[**22/39**: C-G => **U**-G]	[**22/39**: C-G => **U**-G] parallel in Capsosiphonaceae excluding *Protomonostroma undulatum* and *Monostroma* = **hPAR 3**
Gloeotilopsis paucicellularis	2	----	**8/11**: U-A => **C-G**	**8/11**: U-A => **C-G** parallel in *Pseudoneochloris marina*, *Gloeotilopsis* sp. M3284 = **PAR 3**, and reversal in Gomontiaceae (C-G => U-A) = **REV 2**
Gloeotilopsis sp. ACOI + *G. sarcinoidea* UTEX 1710	2	----	**7/12**: U-A => **C-G**	**7/12**: U-A => **C-G** parallel in *Pseudoneochloris marina*, *Mono-stroma* = **PAR 1**

Gloeotilopsis sp. ACOI	4	**6/13**: A-U => **C-G**<br>**9/10**: C-G => **U-A**	----	----
Gloeotilopsis sarcinoidea UTEX 1710	5	----	**54/121**: U-G => **A-U**<br><br>[**55/120**: U-A **=>** U-**G**]	**54/121**: U-G => **A-U**, **A-U** convergent in Kornmanniaceae + Bolbocoleonaceae, *Collinsiella* (U-A => A-U) = **CONV 2**<br>[**55/120**: U-A => U-**G**] parallel in *Ulva* + *Percursaria* + *Ulvaria*, *Monostroma* excluding *M*. *grevillei* AJ000205 = **hPAR 6**
Gloeotilopsis sp. M3284	2	----	**8/11**: U-A => **C-G**	**8/11**: U-A => **C-G** parallel in *Pseudoneochloris marina*, *Gloeotilopsis paucicellularis*= **PAR 3**, and reversal in Gomontiaceae (C-G => U-A) = **REV 1**
'*Pseudendoclonium basiliense*'	3	[**26/35**: C-G **=>** **U-**G]	----	----
