# Supplementary material for: A close-up view on ITS2 evolution and speciation - a case study in the Ulvophyceae (Chlorophyta, Viridiplantae)
Source: BMC Evol Biol. 2011 Sep 20;11:262. doi: 10.1186/1471-2148-11-262 (PMC3225284; doi:10.1186/1471-2148-11-262)
Supplement: Additional file 4 — List of all substitutions of ITS2 base pairs during the evolution of the Ulvales. For nucleotide numbers, see Figure 1A. CBCs (blue) and hCBCs (red) were classified into non-homoplasious (NHS) and homoplasious character changes (HS, categorized into parallelisms, convergences, and reversals; or further explanation, see Additional file 3). For every pair, the likely plesiomorphic character status within the Ulvales is given. Moreover, non-compensating base changes are listed here, that involve a pair ⇔ unpair conversion. The conserved regions of helices 2 and 3 were depicted in gray shades. [file 1471-2148-11-262-S4.PDF]

List of all substitutions of ITS2 base pairs during the evolution of the Ulvales.

Helix 1

6/13: **A-U** - plesiomorphic status in the Ulvales
6/13: A-U => **G-C** in *Ulva reticulata* clade; **NHS**
6/13: A-U => **C-G** in *Gloeotilopsis* sp. ACOI; **NHS**

7/12: **C-G** - plesiomorphic status in the Ulvales
7/12: U-A => **C-G** in *Pseudoneochloris marina*; **HS** (PAR 1)
7/12: U-A => **C-G** in *Monostroma*; **HS** (PAR 1)
7/12: U-A => **C-G** in *Gloeotilopsis* sp. ACOI + *G. sarcinoidea* UTEX 1710; **HS** (PAR 1)
7/12: C-G => C×**A** in *Percursaria percursa* AY016305
7/12: C-G => **A×C** in *Acrochaete* + ‘*Umbraulva japonica*’, futher changes in all taxa excluding ‘*Umbraulva japonica*’
7/12: C-G => C×**A** in Capsosiphonaceae + Gomontiaceae
7/12: ≈3× N×N => N-**N** (the total number cannot be estimated precisely due to their high substitution frequency) as an example:
7/12: C×A => **U**-A in *Protomonostroma undulatum*

8/11: **C-G** - plesiomorphic status in the Ulvales
8/11: C-G => **G-C** in *Ulva*; **NHS** - futher changes in *U. lactuca* clade, *U. muscoides*
8/11: C-G => **U-A** in *Percursaria*; **HS** (PAR 2)
8/11: C-G => **U-A** in Gomontiaceae; **HS** (PAR 2)
8/11: U-A => **C-G** in *Pseudoneochloris marina*; **HS** (PAR 3; REV 1)
8/11: U-A => **C-G** in *Gloeotilopsis* sp. M3284; **HS** (PAR 3; REV 2)
8/11: U-A => **C-G** in *Gloeotilopsis paucicellularis*; **HS** (PAR 3)
8/11: G-C => G-**U** in *Ulva muscoides*; **NHS**
8/11: U-A => U-**G** in Ulvales sp. EF595507 + Ulvales sp. EF595508 + Ulvales sp. EF595509; **NHS**
8/11: ≈8× N-N => N×N (the total number cannot be estimated precisely due to their high substitution frequency) as an example:
8/11: C-G => **U**×**U** in *Acrochaete heteroclada*
8/11: ≈3× N×N => N-**N** (the total number cannot be estimated precisely due to their high substitution frequency) as an example:
8/11: A×C => A-**U** in *Kornmannia*

9/10: **C-G** - plesiomorphic status in the Ulvales
9/10: C-G => **G-C** in ‘*Blidingia minima*’ EF595512; **NHS**
9/10: C-G => **U-A** in *Gloeotilopsis* sp. ACOI; **NHS**
9/10: ≈5× N-N => **N×N** (the total number cannot be estimated precisely due to their high substitution frequency) as an example:
9/10: C-G => C×**C** in *Collinsiella*

Helix 2

20/41: **G-U** - plesiomorphic status in the Ulvales
20/41: G-U => G-**C** in *Gloeotilopsis* clade; **HS** (hPAR 1)
20/41: G-U => G-**C** in Bolbocoleonaceae; **HS** (hPAR 1)

21/40: **G-C** - plesiomorphic status in the Ulvales
21/40: G-C => **A-U** in *Acrochaete viridis*; **NHS**
21/40: G-C => G-**U** in Bolbocoleonaceae; **HS** (hPAR 2)
21/40: G-C => G-**U** in *Acrochaete heteroclada*; **HS** (hPAR 2)

22/39: **C-G** - plesiomorphic status in the Ulvales
22/39: C-G => **U**-G in *Gloeotilopsis* clade excluding *Gloeotilopsis* sp. M3284; **HS** (hPAR 3)
22/39: C-G => **U**-G in Capsosiphonaceae excluding *Protomonostroma undulatum*; **HS** (hPAR 3)
22/39: C-G => **U**-G in *Monostroma*; **HS** (hPAR 3)

23/38: **C-G** - plesiomorphic status in the Ulvales
23/38: C-G => **U-A** in *Kornmannia*; **NHS**
23/38: C-G => **G-C** in *Collinsiella*; **NHS**

Helix 2

<u>26/35: **C-G** - plesiomorphic status in the Ulvales</u>

26/35: C-G => **U-G** in *'Pseudendoclonium basiliense'*; **NHS**

<u>27/34: **C-G** - plesiomorphic status in the Ulvales</u>

27/34: C-G => **U-G** in *Monostroma arcticum* + *M. nitidum*; **HS** — **hPAR 4**

27/34: C-G => **U-G** in Capsosiphonaceae; **HS** — **hPAR 4**

27/34: C-G => **U-G** in *Ulva tanneri*; **HS** — **hPAR 4**

<u>28/33: **C-G** - plesiomorphic status in the Ulvales</u>

28/33: C-G => **G-C** in Kornmanniaceae + Bolbocoleonaceae; **NHS**

<u>29/32: **A-U** - plesiomorphic status in the Ulvales</u>

29/32: A-U => **G-C** in Ulvaceae; **NHS**

<u>30/31: **G-C** - plesiomorphic status in the Ulvales</u>

30/31: G-C => **C-G** in Gomontiaceae excluding *Chamaetrichon capsulatum* and *'Pseudendoclonium basiliense'*; **HS** — **PAR 4**

30/31: G-C => **C-G** in *Acrosiphonia*; **HS** — **PAR 4**

30/31: C-G => **U-A** in *Monostroma*; **NHS**

30/31: G×G => **U**-G in *'Pseudendoclonium basiliense'*

30/31: G-C => G×**A** in *Chamaetrichon capsulatum*

30/31: G-C => G×**A** in *Capsosiphon groelandicus*

Helix 3

<u>53/122: **G-C** - plesiomorphic status in the Ulvales</u>

53/122: G-C => **A-U** in Gomontiaceae (marine/ brackish) + *Ulothrix zonata*; **NHS**

<u>54/121: **U-A** - plesiomorphic status in the Ulvales</u>

54/121: U-A => **A-U** in Kornmanniaceae + Bolbocoleonaceae; **HS** — **PAR 5**; **CONV 2**

54/121: U-A => **A-U** in *Collinsiella*; **HS** — **PAR 5**; **CONV 1**

54/121: U-G => **A-U** in *Gloeotilopsis sarcinoidea* UTEX 1710; **HS** — **CONV 1**; **CONV 2**

54/121: U-A => **C-G** in Ulvaceae; **NHS**

54/121: U-A => U-**G** in *Gloeotilopsis* clade; **HS** — **hPAR 5**

54/121: U-A => U-**G** in *'Blidingia minima'* AJ000206; **HS** — **hPAR 5**

54/121: U-A => **C**×A in *'Blidingia minima'* EF595512

<u>55/120: **U-A** - plesiomorphic status in the Ulvales</u>

55/120: U-A => **A-U** in *Acrosiphonia*; **HS** — **CONV 3**

55/120: G-C => **A-U** in *Kornmannia*; **HS** — **CONV 3**

55/120: U-A => **G-C** in Kornmanniaceae; **HS** — **PAR 6**

55/120: U-A => **G-C** in *'Blidingia minima'*; **HS** — **PAR 6**

55/120: U-A => U-**G** in *Gloeotilopsis sarcinoidea* UTEX 1710; **HS** — **hPAR 6**

55/120: U-A => U-**G** in *Monostroma* excluding *M. grevillei* AJ000205; **HS** — **hPAR 6**

55/120: U-A => U-**G** in *Ulva* + *Percursaria* + *Ulvaria*; **HS** — **hPAR 6**

55/120: U-G => U-**A** in *Ulva* californica AJ234315; **HS** — **hREV 1**

55/120: U-A => U×**C** in Bolbocoloenaceae

55/120: U-A => **G**×A in *Pseudoneochloris marina*

55/120: U-A => U×**U** in *Capsosiphon groelandicus*

55/120: U-A => U×**U** in *'Umbraulva japonica'*

<u>57/119: **A×C** - plesiomorphic status in the Ulvales</u>

57/119: ≈6× N×N => N-**N** (the total number cannot be estimated precisely due to their high substitution frequency) as an example:

57/119: A×C => **G**-C in *Pseudoneochloris marina*

Helix 3

58/118: **C×A** or **A×A** - plesiomorphic status in the Ulvales

58/118: C-G => **U**-G in *Ulva*; **HS** - futher changes in *Ulva lactuca* clade + *U. muscoides* + *U. taeniata* + *U. reticulata* clade + *U. californica* AB280867 + *Ulva* sp. EU933983

58/118: U-G => **C**-G in *Ulva lactuca* clade + *U. muscoides* + *U. taeniata* + *U. reticulata* clade + *U. californica* AB280867 + *Ulva* sp. EU933983; **HS** - futher change in *U. californica* AB280867

58/118: C-G => **U**-G in *U. californica* AB280867; **HS**

hREV 2

hPAR 7

hREV 3

58/118: C×C => **U-G** in *Kornmannia*

58/118: C×A => C-**G** in Ulvaceae

58/118: A×A => **C-G** in *Pseudoneochloris marina*

Helix 3

59/117: **G-C** - plesiomorphic status in the Ulvales

59/117: G-C => G-**U** in *Acrochaete* sp. EF595429; **HS**

59/117: G-C => G-**U** in *Ulva tanneri*; **HS**

59/117: G-C => G-**U** in *Ulva flexuosa* + *Ulva californica* AJ234315; **HS**

59/117: G-C => G-**U** in *Ulva linza* AJ012276, AJ000203 + *U. prolifera* + *U. lactuca* clade + *U. muscoides* + *U. taeniata* + *U. reticulata* clade + *U. californica* AB280867 + *Ulva* sp. EU933983; **HS** - futher changes in *U. taeniata, U. fasciata* + *U. pertusa*

hPAR 8

hREV 4

hREV 5

59/117: G-U => G-**C** in *Ulva fasciata* + *Ulva pertusa*; **HS**

59/117: G-U => G-**C** in *Ulva taeniata*; **HS**

hPAR 9

59/117: ≈2× N-N => **N×N** (the total number cannot be estimated precisely due to their high substitution frequency) as an example:

59/117: G-C => G×**G** in *Pseudoneochloris marina*

59/117: G×G => G-**U** in *Kornmannia*

60/116: **C-G** - plesiomorphic status in the Ulvales

60/116: C-G => **U**-G in *Blidingia chadefaudii*; **NHS**

60/116: C-G => C×**C** in ‘*Umbraulva japonica*’

60/116: C-G => C×**U** in *Acrochaete* sp. EF595429

60/116: C-G => C×**U** in Bolbocoleonaceae

Helix 3

62/114: **U×U** or **U-G** - plesiomorphic status in the Ulvales

62/114: U-G => **C**-G in Kornmanniaceae; **HS**

62/114: U-G => **C**-G in Ulvaceae; **HS**

hPAR 10

62/114: ≈4× N-N => **N×N** (the total number cannot be estimated precisely due to their high substitution frequency) as an example:

62/114: C-G => C×**C** in *Ulva stenophylla*

62/114: U×U => **C-G** in Gomontiaceae marine/brackish

62/114: U×U => U-**A** in ‘*Blidingia minima*’

63/113: **G-C** - plesiomorphic status in the Ulvales

63/113: G-C => G-**U** in *Ulvales* EF595507; **HS**

63/113: G-C => G-**U** in *Acrochaete* sp. EF595429; **HS**

63/113: G-C => G-**U** in *Ulva reticulata* clade; **HS**

hPAR 11

63/113: G-C => G×**G** in *Acrochaete* sp. EF595413, *Acrochaete* sp. EF595372, ‘*Umbraulva japonica*’

67/110: **C-G** - plesiomorphic status in the Ulvales

67/110: C-G => **U-A** in *Kornmannia*; **NHS**

Helix 3

<u>68/109: **U-G** - plesiomorphic status in the Ulvales</u>
68/109: U-G => **C**-G in Bolbocoleonaceae; **NHS**
68/109: U-G => **A**×G in Ulvaceae

<u>72/108: **G-C** - plesiomorphic status in the Ulvales</u>
72/108: G-C => G-**U** in Capsosiphonaceae + Gomontiaceae; **HS**
72/108: G-C => G-**U** in *Kornmannia*; **HS**
72/108: G-C => G-**U** in *Ulva*; **HS** - futher changes in *U. muscoides* + *U. taeniata*
hPAR 12
72/108: G-U => G-**C** in *Ulva muscoides* + *Ulva taeniata*; **HS**
hREV 6

<u>73/107: **G-C** - plesiomorphic status in the Ulvales</u>
73/107: G-C => G-**U** in *Acrochaete repens*; **NHS**

<u>74/106: **C-G** - plesiomorphic status in the Ulvales</u>
74/106: C-G => **U-A** in *Acrochaete heteroclada*; **NHS**

<u>75/105: **A-U** - plesiomorphic status in the Ulvales</u>
75/105: A-U => **C-G** in Ulvaceae; **NHS**
75/105: A-U => A×**C** in Kornmanniaceae + Bolbocoleonaceae
75/105: A×C => A-**U** in *Kornmannia*

Helix 3

<u>77/103: **C-G** - plesiomorphic status in the Ulvales</u>
77/103: C-G => **G**×G in *Pseudoneochloris marina*

<u>78/102: **U-A** - plesiomorphic status in the Ulvales</u>
78/102: U-A => **G-C** in Kornmanniaceae; **HS**
78/102: U-A => **G-C** in Ulvaceae; **HS**
PAR 7
78/102: G-C => G-**U** in '*Umbraulva japonica*', *Acrochaete* sp. EF595429, *Acrochaete* sp. EF595413, *Acrochaete* sp. EF595372; **NHS**

<u>79/101: **G-C** - plesiomorphic status in the Ulvales</u>
79/101: G-C => **A-U** in *Kornmannia*; **NHS**
79/101: G-C => **A**×C in *Ulva, Ulvaria, Percursaria*

<u>80/100: **C-G** - plesiomorphic status in the Ulvales</u>
80/100: C-G => **U**-G in *Acrochaete* + '*Umbraulva japonica*'; **NHS**

Helix 3

<u>84/97: **G-C** - plesiomorphic status in the Ulvales</u>
84/97: G-C => G×**G** in Ulvaceae
84/97: G×G => G-**U** in *Ulva tanneri*
84/97: G×G => G-**U** in *Ulva prolifera, U. linza* AJ012276, AJ000203

<u>90/93: **A-U** - plesiomorphic status in the Ulvales</u>
90/93: A-U => **G-C** in Gomontiaceae (marine/ brackish); **NHS**
90/93: A-U => **G**-U in '*Umbraulva japonica*', *Acrochaete* sp. EF595429, *Acrochaete* sp. EF595413, *Acrochaete* sp. EF595372; **NHS**

<u>91/92: **G-C** - plesiomorphic status in the Ulvales</u>
91/92: G-C => G-**U** in Gomontiaceae (marine/ brackish); **HS**
91/92: G-C => G-**U** in *Urospora* sp. AY476812 + *Urospora wormskioldii*; **HS**
hPAR 13

Helix 4

<u>125/129: **G-C** - plesiomorphic status in the Ulvales</u>
125/129: G-C => **A-U** in Gomontiaceae (marine/ brackish); **NHS**
125/129: G-C => G-**U** in *Percursaria*; **NHS**

<u>126/128: **G-C** - plesiomorphic status in the Ulvales</u>
126/128: G-C => G-**U** in Kornmanniaceae + Bolbocoleonaceae; **HS**
126/128: G-C => G-**U** in Gomontiaceae (marine/ brackish) + *Ulothrix zonata*; **HS**
hPAR 14

Helix 4

126/128: G-C => G-**U** in *Acrochaete* + ‘*Umbraulva japonica*’; **HS**

126/128: G-C => G-**U** in *Ulva muscoides*; **HS**

126/128: G-C => G-**U** in *Ulva lactuca* clade; **HS**

hPAR 14
