# Supplementary material for: A close-up view on ITS2 evolution and speciation - a case study in the Ulvophyceae (Chlorophyta, Viridiplantae)
Source: BMC Evol Biol. 2011 Sep 20;11:262. doi: 10.1186/1471-2148-11-262 (PMC3225284; doi:10.1186/1471-2148-11-262)

Compensatory base changes distributed over conserved regions of helices 2 and 3 of ITS2 in the Ulvales

Legend:

Universal positions:

N : position 100% conserved

N/● : position with only 1 change

N/● : position with 2-5 changes

Nonuniversal positions:

● : position with >6 changes

● : expansion segments

N : specific insertions (IUPAC-symbols used)

▪▪▪ : base pairs in the majority of taxa

Nucleotide symbols: dominant character state in 70-100% of the Ulvales

Circles: position below 70% majority rule consensus

Compensatory base changes:

N→N : Compensatory base changes (CBCs) in coserved regions of helices 2 and 3

NHS : non-homoplasious compensatory base changes

HS : homoplasious compensatory base changes

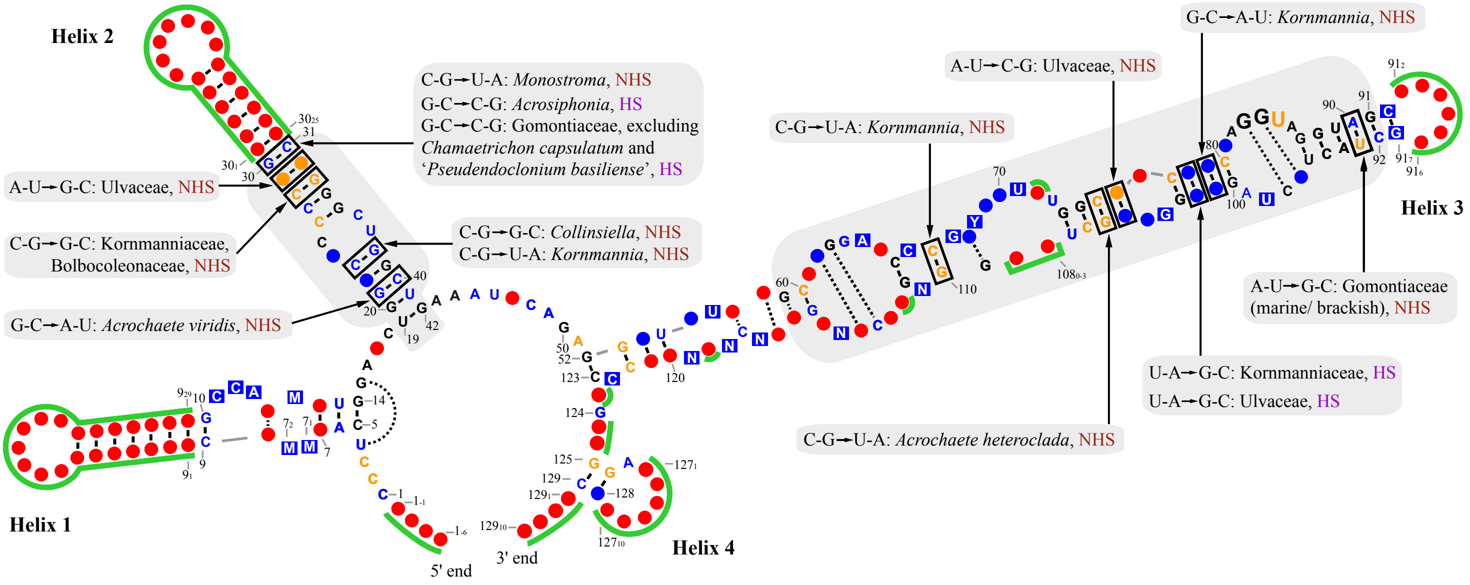

Supplement: Additional file 5 — Compensatory base changes distributed over conserved regions of helices 2 and 3 of ITS2 in the Ulvales. All 15 compensatory base changes found in conserved regions of helices 2 and 3 (H2+3_CBCs) were mapped on the consensus secondary structure model of ITS2 in the Ulvales. Comments refer either to their non-homoplasious (NHS) or to homoplasious (HS) status. For further information on universal/non-universal positions see Figure 1. [file 1471-2148-11-262-S5.PDF]
