# Supplementary material for: A close-up view on ITS2 evolution and speciation - a case study in the Ulvophyceae (Chlorophyta, Viridiplantae)
Source: BMC Evol Biol. 2011 Sep 20;11:262. doi: 10.1186/1471-2148-11-262 (PMC3225284; doi:10.1186/1471-2148-11-262)
Supplement: Additional file 7 — Strain designations, origins and accession-numbers of nuclear-encoded ITS2 rRNA 86 strains of the Ulvales. Newly determined sequences are in bold. An asterisk (*) indicates authentic cultures. [file 1471-2148-11-262-S7.DOC]

| **Taxon and strain designation** | **Isolator/Collector, isolation data** | **Accession-number** |
| --- | --- | --- |
| **Kornmanniaceae** |  |  |
| *Blidingia chadefaudii* (Feldmann) Bliding | I. H. Tan, J. Blomster, G. Hansen, E. Leskinen, C. A. Maggs, D. G. Mann, H. J. Sluiman & M. J. Stanhope, specimen voucher: F116624, other information unknown | AJ012309 |
| *Blidingia dawsonii* (Hollenberg & Abbott) Lindstrom, Hanic & Golden | S. C. Lindstrom, 2003, Canada, Seppings Island, Barkley Sound, British Columbia, isolate: 5, specimen voucher: A84927 (UBC) | DQ001138 |
| *Kornmannia leptoderma* (Kjellman) Bliding | Q. Su, R. Luan & L. An, other information unknown | AF415168 |
| *Kornmannia leptoderma* (Kjellman) Bliding | B. Rinkel, 2004, UK, West Sutherland, Portskerra, Melvich, isolate: e070ps | EF595513 |
| *Ulvales* sp. 3-BER-2007ee/ B33cm3 | B. Rinkel, 2003, UK, North Devon, Combe Martin Bay, epi-/endophyte of *Mastocarpus stellatus*, isolate: B33cm3 | EF595495 |
| **Bolbocoleonaceae** |  |  |
| *Bolbocoleon piliferum* Pringsheim | B. Rinkel, 2004, UK, North Aberdeenshire, Rosehearty, endophyte of *Chorda filum*, isolate: e094rh | EF595481 |
| **Ulvaceae** |  |  |
| **Ulva-clade** |  |  |
| *Ulva armoricana* **Dion, de Reviers & Coat** | N. Yokoyama, 2004, Japan, Hokkaido, Hakodate, isolate: NY054, specimen voucher: SAP:100109 | AB275824 |
| *Ulva armoricana* **Dion, de Reviers & Coat** | N. Yokoyama, 2004, Japan, Hokkaido, Hakodate, isolate: NY057, specimen voucher: SAP:100109 | AB275825 |
| *Ulva californica* Wille | S. Shimada, T. Hanyuda, T. Suzuki & H. Kawai, 2005, Japan, Aichi, Mikawa Bay, Tahara, isolate: MK-77 | AB280867 |
| *Ulva californica* Wille | I. H. Tan, J. Blomster, G. Hansen, E. Leskinen, C. A. Maggs, D. G. Mann, H. J. Sluiman & M. J. Stanhope, isolate: OO0101, other information unknown | AJ234315 |
| *Ulva fasciata* Delile | S. Shimada, M. Hiraoka, S. Nabata, M. Iima & M. Masuda, year unknown, Japan, Kochi, Usa, specimen voucher: SAP:095075 | AB097663 |
| *Ulva fenestrata* Postels & Ruprecht | I. H. Tan, J. Blomster, G. Hansen, E. Leskinen, C. A. Maggs, D. G. Mann, H. J. Sluiman & M. J. Stanhope, isolate: OM0101, other information unknown | AJ234316 |
| *Ulva flexuosa* Wulfen | I. H. Tan, J. Blomster, G. Hansen, E. Leskinen, C. A. Maggs, D. G. Mann, H. J. Sluiman & M. J. Stanhope, isolate: E45, other information unknown, in NCBI under synonym *Enteromorpha flexuosa* | AJ234306 |
| *Ulva laetevirens* Areschoug | L. G. K. Kraft, G. T. Kraft & R. F. Waller, 2007, Australia, Port Adelaide, SA, specimen voucher: LK-049 | EU933989 |
| *Ulva lactuca* Linnaeus | I. H. Tan, J. Blomster, G. Hansen, E. Leskinen, C. A. Maggs, D. G. Mann, H. J. Sluiman & M. J. Stanhope, isolate: SR0103, other information unknown | AJ000208 |
| *Ulva linza* Linnaeus | I. H. Tan, J. Blomster, G. Hansen, E. Leskinen, C. A. Maggs, D. G. Mann, H. J. Sluiman & M. J. Stanhope, isolate: SY0101, other information unknown, in NCBI under synonym *Enteromorpha linza* | AJ000203 |
| *Ulva linza* Linnaeus | E. Leskinen & P. Pamilo, isolate: E24, specimen voucher: A00278, other information unknown, in NCBI under synonym *Enteromorpha ahlneriana* | AJ012276 |
| *Ulva muscoides* Clemente ex Schousboe | J. Blomster, C. A. Maggs & M. J. Stanhope, isolate: 613, other information unknown, in NCBI under synonym *Enteromorpha muscoides* | AF127168 |
| *Ulva muscoides* Clemente ex Schousboe | I. H. Tan, J. Blomster, G. Hansen, E. Leskinen, C. A. Maggs, D. G. Mann, H. J. Sluiman & M. J. Stanhope, isolate: B612, other information unknown, in NCBI under synonym *Enteromorpha muscoides* | AJ234307 |
| *Ulva muscoides* Clemente ex Schousboe | J. Blomster, C. A. Maggs & M. J. Stanhope, isolate: 601, other information unknown, in NCBI under synonym *Enteromorpha muscoides* | AF127165 |
| *Ulva ohnoi* **Hiraoka & Shimada in Hiraoka, Shimada, Uenosono & Masuda** | M. Hiraoka, S. Shimada, M. Uenosono & M. Masuda, Japan,Okinawa, Naha, specimen voucher: SAP:095160, other information unknown | AB116029 |
| *Ulva pertusa* Kjellman | I. H. Tan, J. Blomster, G. Hansen, E. Leskinen, C. A. Maggs, D. G. Mann, H. J. Sluiman & M. J. Stanhope, isolate: K0101, other information unknown | AJ234321 |
| *Ulva prolifera* **(Forsskål) de Candolle in Lamarck** | X. Zhang, N. Ye, Y. Mao, Z. Zhuang & Q. Wang, other information unknown | FJ026732 |
| *Ulva prolifera* **(Forsskål) de Candolle in Lamarck** | S. Shen, Z. Lin & B. Zhang, strain: ShanT 2, other information unknown | HM047555 |
| *Ulva reticulata* Forsskål | S. Shimada, M. Hiraoka, S. Nabata, M. Iima & M. Masuda, year unknown, Philippines, Cebu Island, specimen voucher: SAP:095077 | AB097665 |
| *Ulva rigida* Agardh | I. H. Tan, J. Blomster, G. Hansen, E. Leskinen, C. A. Maggs, D. G. Mann, H. J. Sluiman & M. J. Stanhope, specimen voucher: SSBO0102, other information unknown | AJ234319 |
| *Ulva scandinavica* Bliding | S. Shimada, M. Hiraoka, S. Nabata, M. Iima & M. Masuda, year unknown, Netherlands, Wadden Sea, strain number by isolator: SAP:095071 | AB097659 |
| *Ulva spinulosa* Okamura & Segawa | S. Shimada, M. Hiraoka, S. Nabata, M. Iima & M. Masuda, year unknown, Japan, Kochi, Fubenhama, specimen voucher: SAP:095078 | AB097666 |
| *Ulva stenophylla* **Setchell & Gardner** | H. S. Hayden, J. Blomster, C. A. Maggs, P. C. Silva, M. J. Stanhope & J. R. Waaland, specimen voucher: WTU344829, other information unknown | AY260569 |
| *Ulva taeniata* **(Setchell) Setchell & Gardner** | H. S. Hayden & J. R. Waaland,isolate: Utae99-17, strain: UWCC MA723, specimen voucher: WTU344833, other information unknown | AY422525 |
| *Ulva tanneri* Hayden & Waaland | L. G. K. Kraft, G. T. Kraft & R. F. Waller, 2006, Australia, Brisbane, QLD, specimen voucher: LK-013 | EU933971 |
| *Ulva* sp. LGKK-2008e/ LK-032 | L. G. K. Kraft, G. T. Kraft & R. F. Waller, 2006, Australia, Queenscliff, VIC, specimen voucher: LK-032 | EU933983 |
| *Acrochaete heteroclada* Correa & Nielsen | B. Rinkel, 2004, UK, Anglesey, Rhosneigr, epi-/endophyte of *Chondrus crispus*, isolate: e289rn | EF595444 |
| *Acrochaete repens* Pringsheim | B. Rinkel, 2004, UK, Westerness, West Port, Kintyre Peninsula, endophyte of *Chorda filum*, isolate: e103wp | EF595436 |
| *Acrochaete repens* Pringsheim | B. Rinkel, 2004, UK, North Aberdeenshire, Rosehearty, endophyte of *Chondrus crispus*, isolate: e282rh | EF595437 |
| *Acrochaete viridis* (Reinke) Nielsen | B. Rinkel, 2004, UK, Kincardineshire, Seagreens, endophyte of *Osmundea osmunda*, isolate: e170sg | EF595455 |
| *Acrochaete* sp. 1-BER-2007/ e058bt | B. Rinkel, 2004, UK, North Northumberland, Berwick-upon-Tweed, epi-/endophyte of *Chondrus crispus*, isolate: e058bt | EF595372 |
| *Acrochaete* sp. 3-BER-2007/ B146cb15 | B. Rinkel, 2004, UK, Pembrokeshire, Castle Beach, epi-/endophyte of *Mastocarpus stellatus*, isolate: B146cb15 | EF595413 |
| *Acrochaete* sp. 4-BER-2007/ e134bt | B. Rinkel, 2004, UK, North Northumberland, Berwick-upon-Tweed, epi-/endophyte of *Mastocarpus stellatus*, isolate: e134bt | EF595429 |
| *Percursaria percursa* (Agardh) Rosenvinge | G. W. Woolcott & R. J. King, other information unknown | AY016305 |
| *Percursaria percursa* (Agardh) Rosenvinge | H. S. Hayden, J. Blomster, C. A. Maggs, P. C. Silva, M. J. Stanhope & J. R. Waaland, isolate: UWCC MA230, other information unknown | AY260570 |
| *Ulvaria fusca* (Ruprecht in Papenfuss) Vinogradova | S. Shimada, M. Hiraoka, S. Nabata, M. Iima & M. Masuda, Japan, Hokkaido, Oshoro, specimen voucher: SAP:095049, other information unknown | AB097637 |
| *Ulvaria obscura* (Areschoug) Bliding | H. S. Hayden, J. Blomster, C. A. Maggs, P. C. Silva, M. J. Stanhope & J. R. Waaland, specimen voucher: WTU344838, other information unknown | AY260571 |
| ‘*Umbraulva japonica*’- likely misidentified | S. Shimada, M. Hiraoka, S. Nabata, M. Iima & M. Masuda, Japan, Shizuoka, Shimoda, specimen voucher: SAP: 095050, other information unknown | AB097638 |
| ‘*Umbraulva japonica*’- likely misidentified | S. Shimada, M. Hiraoka, S. Nabata, M. Iima & M. Masuda, Japan, Chiba, Tatemana, specimen voucher: SAP:095051, other information unknown | AB097639 |
| **Capsosiphonaceae excluding *Pseudoneochloris marina*** |  |  |
| *Acrosiphonia arcta* (Dillwyn) Gain | A. V. Sussmann, B. K. Mable, R. E. deWreede & M. L. Berbee, isolate: Sk/BI, other information unknown | AF019256 |
| *Acrosiphonia coalita* (Ruprecht) Scagel, Garbary, Golden & Hawkes | A. V. Sussmann, other information unknown | AF047682 |
| ***Acrosiphonia* sp**. SAG 127.80 | P. Kornmann, year unknown, Germany, Helgoland, marine habitat | HE575898 |
| ‘*Blidingia minima*’- likely misidentified | B. Rinkel, 2003, UK, North Devon, isolate: B36cm1 | EF595512 |
| ‘*Blidingia minima*’- likely misidentified | I. H. Tan, J. Blomster, G. Hansen, E. Leskinen, C. A. Maggs, D. G. Mann, H. J. Sluiman & M. J. Stanhope, isolate: SMU0101, other information unknown | AJ000206 |
| *Capsosiphon groenlandicus* (Agardh) Vinogradova | S. C. Lindstrom, 2004, USA, latitude and longitude: 53.8939N/166.5432W, other information unknown | DQ821514 |
| *Chlorothrix* sp. C167 | S. C. Lindstrom & L. A. Hanic, North America, other information unknown | AY653740 |
| *Protomonostroma undulatum* (**Wittrock)** *Vinogradova* | S. C. Lindstrom, 2003, USA, Alaska, Katmai National Park, Shaw Island, drift, latitude and longitude: 59.1444N/153.5167W | DQ821517 |
| *Ulothrix* sp. X1 | S. C. Lindstrom, 2004, USA, latitude and longitude: 53.8939N/166.5432W | DQ821515 |
| *Ulothrix* sp. X3 | S. C. Lindstrom, 2004, USA, Alaska, Amaknak Island, mid-intertidal boulders, , latitude and longitude: 53.8939N/166.5432W | DQ821516 |
| ‘*Ulvales*’ sp. 6-BER-2008/ B86cm1 | B. Rinkel, 2003, UK, North Devon, Combe Martin Bay, epi-/endophyte of *Mastocarpus stellatus*, isolate: B86cm1 | EF595507 |
| ‘*Ulvales*’ sp. 6-BER-2007ee/ B3sn2 | J. Brodie, 2003, UK, East Kent, Sheerness, epi-/endophyte of *Chondrus crispus*, isolate: B3sn2 | EF595508 |
| ‘*Ulvales*’ sp. 6-BER-2007ee/ e009wb | B. Rinkel, 2004, UK, South Northumberland, Whitburn Bay, epi-/endophyte of *Mastocarpus stellatus*, isolate: e009wb | EF595509 |
| ‘*Ulvales*’ sp. 6-BER-2007ee/B5mr2 | J. Brodie, 2003, UK, East Kent, Minster, epi-/endophyte of *Chondrus crispus*, isolate: B5mr2 | EF595510 |
| ‘*Ulvales*’ sp. 6-BER-2007ee/ B56sm11 | B. Rinkel, 2003, UK, South Devon, Sidmouth, epi-/endophyte of *Osmundea* sp., isolate: B56sm11 | EF595511 |
| *Urospora neglecta* (Kornmann) Lokhorst & Trask | S. C. Lindstrom & L. A. Hanic, North America, strain: Seward 8, isolate: Uneg121, other information unknown | AY476821 |
| *Urospora wormskioldii* (Mertens ex Hornemann) Rosenvinge | S. C. Lindstrom & L. A. Hanic, North America, strain: San Simeon, isolate: UW117, other information unknown | AY476817 |
| *Urospora* sp. AB58 | S. C. Lindstrom & L. A. Hanic, North America, strain number by isolator: Aberdeen 10, isolate: AB58, other information unknown | AY476812 |
| *Urospora* sp. U122 | S. C. Lindstrom & L. A. Hanic, North America, strain: Pacific Viking, isolate: U122, other information unknown | AY476820 |
| uncultured *Urospora* | I. Moro, E. Negrisolo, A. Callegaro & C. Andreoli, Antarctica:Terra Nova Bay, Ross Sea, environmental sample | AJ626846 |
| ***Pseudoneochloris marina*** **Watanabe, Himizu, Lewis, Floyd & Fuerst CCMP 257** | R. Guillard, 1955, North America, USA, Connecticut, Milford, tank, seawater | HE575897 |
| **Gomontiaceae** |  |  |
| **Gomontiaceae (marine/ brackish)** |  |  |
| *Collinsiella tuberculata* Setchell & Gardner | C. J. O'Kelly, B. Wysor & W. K. Bellows, year unknown, Canada, Botanical Beach, Vancouver Island, BC, sloping rocky bench, midtidal zone, specimen voucher: WA4-14 | AY198124 |
| *Collinsiella tuberculata* Setchell & Gardner | C. J. O'Kelly, B. Wysor & W. K. Bellows, year unknown, USA, Cattle Point, San Juan Island, WA, high intertidal tide pools, specimen voucher: WA3 | AY198125 |
| *Monostroma angicava* Kjellman | Q. Su, R. Luan & L. An, isolate: AST20009018, other information unknown | AF415173 |
| *Monostroma arcticum* **Wittrock** | Q. Su, R. Luan & L. An, isolate: AST20009010, other information unknown | AF415171 |
| *Monostroma grevillei* **(Thuret) Wittrock** | C. A. Maggs, UK, other information unknown | GU062560 |
| *Monostroma grevillei* **(Thuret) Wittrock** | I. H. Tan & H. J. Sluiman, strain number by isolator: SY02b02, other information unknown | AJ000205 |
| *Monostroma grevillei* **(Thuret) Wittrock** | Q. Su, R. Luan & L. An, isolate: AST20009017, other information unknown | AF428050 |
| *Monostroma nitidum* **Wittrock** | G. W. Woolcott & R. J. King, other information unknown | AY026917 |
| **Gomontiaceae (freshwater/ soil)** |  |  |
| ***Chamaetrichon capsulatum*** Tupa UTEX 1918* | D. D. Tupa, 1969, USA, Texas, Sam Houston National Forest, Pallavicinia lyellii, Double Lake, strain: DDT-8 | HE575896 |
| *Pseudendoclonium basiliense* Vischer UTEX 2593* | W. Vischer,1923, Switzerland, Basel, freshwater | Z47996 |
| *Ulothrix zonata* (Weber & Mohr) Kützing SAG 38.86 | M. Uhde, 1984, Czech Republic, North Bohemia, brook in Jeseníky Mts., freshwater | Z47999 |
| ***Gloeotilopsis*-clade** |  |  |
| ***Chamaetrichon capsulatum*** Tupa SAG 8.90 | H. J. Sluiman, 1985, Czech Republic, near Třeboň, sandy soil at a lake, strain: CS 32-2 | HE575891 |
| ***Chlorosarcinopsis minor*** (Gerneck) Herndon ACOI 593 | J. Paiva, 1992, Portugal, S. Tomé e Príncipe, between S. Luís and Chamiço, forest pond | HE575894 |
| ***Gloeotilopsis paucicellularis*** (Vischer) Friedl SAG 463-1* | W. Vischer, 1930, Switzerland, Bot. Gard. Univ. Basel, stagnant water, strain: 68 | HE575889 |
| ***Gloeotilopsis paucicellularis*** (Vischer) Friedl M3283 | A. Lukešová, 1987, Czech Republic, near Chelčice, South Bohemia, soil from arable field, cambisol, strain: ISBAL 177 | HE575887 |
| ***Gloeotilopsis planctonica*** Iyengar & Philipose SAG 29.93 | H. J. Sluiman, 1985, Czech Republic, Suchdol/Třeboň, soil from shore of a lake, strain: 31-1 | HE575890 |
| ***Gloeotilopsis sarcinoidea*** (Groover & Bold) Friedl UTEX 1710* | R. D. Groover, 1969, USA, Texas, Bastrop State Park, soil, strain: RDG-34 | HE575895 |
| ***Gloeotilopsis* sp.** M3284 | A. Lukešová, 1997, Brazil, Sao Carlos, central part of Sao Paulo State, sugarcane field, lateritic soil, strain: ISBAL 1052 | HE575888 |
| ***Helicodictyon planctonicum*** (Whitford) Whitford & Schumacher UTEX 1570 | P. Biebel, 1965, USA, Pennsylvania, Mount Holy Springs, flooded ore pit, strain: 5 | HE575892 |
| ***Protoderma sarcinoidea*** (Kützing) ACOI 592 | J. Paiva, 1992, Portugal, S. Tomé e Príncipe, Diogo Vaz, mud, strain: 92/14 | HE575893 |
