# Supplementary material for: A close-up view on ITS2 evolution and speciation - a case study in the Ulvophyceae (Chlorophyta, Viridiplantae)
Source: BMC Evol Biol. 2011 Sep 20;11:262. doi: 10.1186/1471-2148-11-262 (PMC3225284; doi:10.1186/1471-2148-11-262)
Supplement: Additional file 8 — Primers used for PCR amplification/sequencing of ITS2 in the nuclear-encoded rRNA operon of the Ulvales. Since a few cultures were contaminated with fungi, PCR reactions were performed with specific reverse primers that mismatched with fungal rDNA sequences (labelled 'exFungi'; specific 3'-positions underlined). [file 1471-2148-11-262-S8.DOC]

| **PCR-primer** | primer sequence (5' to 3') |
| --- | --- |
| 18S_EAF3_forw | TCGACAATCTGGTTGATCCTGCCAG |
| 28S_ITS055_rev | CTCCTTGGTCCGTGTTTCAAGACGGG |
| Viridi28S_D8_rev_exFungi | CCAGAGTTTCCTCTGGCTTCNCCCTGC |
| Viridi28S_D10r_rev_exFungi | CGATTAGTCTTTCGCCCCTATACCCAAGTC |
| **Sequencing-primer** |  |
| Sequ_18S_1400_forw | CTGCCCTTTGTACACACCGCCCGTC |
| Sequ_5.8S_ITS03_forw | CGATGAAGAACGYAGCGA |
| Sequ_28S_B22_rev | CTTTCCCTCAYRGTACTTGTTYGC |
